# Air quality impacts of crop residue burning in India and mitigation alternatives

**DOI:** 10.1038/s41467-022-34093-z

**Published:** 2022-11-14

**Authors:** Ruoyu Lan, Sebastian D. Eastham, Tianjia Liu, Leslie K. Norford, Steven R. H. Barrett

**Affiliations:** 1 Laboratory for Aviation and the Environment, Massachusetts Institute of Technology, Cambridge, MA 02139 USA; 2 School of Architecture and Planning, Massachusetts Institute of Technology, Cambridge, MA 02139 USA; 3 MIT Joint Program on the Science and Policy of Global Change, Massachusetts Institute of Technology, Cambridge, MA 02139 USA; 4 Department of Earth and Planetary Sciences, Harvard University, Cambridge, MA 02138 USA

**Keywords:** Environmental impact, Environmental impact, Sustainability, Agriculture

## Abstract

Crop residue burning contributes to poor air quality and imposes a health burden on India. Despite government bans and other interventions, this practice remains widespread. Here we estimate the impact of changes in agricultural emissions on air quality across India and quantify the potential benefit of district-level actions using an adjoint modeling approach. From 2003 to 2019, we find that agricultural residue burning caused 44,000–98,000 particulate matter exposure-related premature deaths annually, of which Punjab, Haryana, and Uttar Pradesh contribute 67–90%. Due to a combination of relatively high downwind population density, agricultural output, and cultivation of residue-intensive crops, six districts in Punjab alone contribute to 40% of India-wide annual air quality impacts from residue burning. Burning two hours earlier in Punjab alone could avert premature deaths up to 9600 (95% CI: 8000–11,000) each year, valued at 3.2 (95% CI: 0.49–7.3) billion US dollars. Our findings support the use of targeted and potentially low-cost interventions to mitigate crop residue burning in India, pending further research regarding cost-effectiveness and feasibility.

## Abstract (web summary)

We quantify the air quality and health impacts of Indian crop residue burning and trace these impacts back to individual burning events by hour and district. We find that small interventions, such as burning one hour earlier in the day, may provide broad public health benefits

## Introduction

**Fig. 1:**
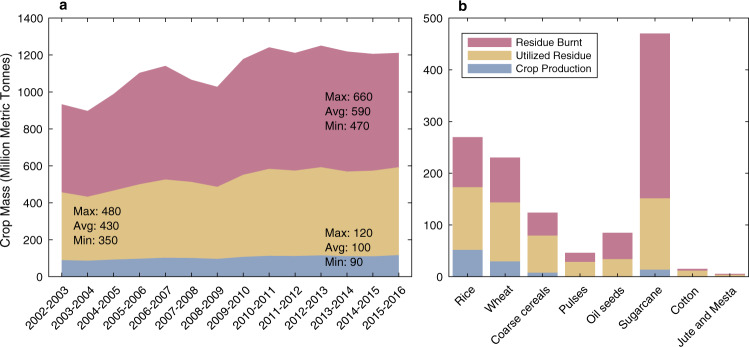
India crop production, utilization and residue by year and crop type. **a** Annual total crop production (blue), utilized residue (yellow), and residue burned (red) in million metric tonnes in India from 2003 to 2016; (**b**) Annual crop production (blue) including crop for food and fiber, utilized residue (yellow) and residue burned (red) by crop type in India, averaged from 2003 to 2016 in million metric tonnes.

Long-term exposure to ambient fine particulate matter (PM_2.5_) is associated with elevated health risks such as respiratory and cardiovascular diseases, resulting in more than four million premature deaths globally each year^1–3^. Of these, 10–25% are estimated to occur in India^3–6^. One source of direct PM_2.5_ emissions responsible for Indian public health impacts is crop residue burning^2–4^. As the second largest worldwide crop producer (Food and Agricultural Organization of the United Nations), India generates ~500 million metric tonnes (MT) of crop residue annually, of which 100 MT is burned (Fig. 1a). The practice of residue burning primarily occurs following the wheat harvest in April-May (pre-monsoon) and the rice harvest in October–November (post-monsoon), and mostly in northwestern India^7,8^. Densely populated areas located downwind of agricultural fires in the Indo-Gangetic Plain (IGP), such as New Delhi, typically experience an annual mean of ambient PM_2.5_ concentration of 50–200 ${{{{{\rm{\mu }}}}}}$g m^−3^ and episodic spikes reaching 200–1200 ${{{{{\rm{\mu }}}}}}$g m^−3^ during burning seasons, exceeding the World Health Organization (WHO) PM_2.5_ guidelines by an order of magnitude (5 ${{{{{\rm{\mu }}}}}}$g m^−3^ annual mean; 15 ${{{{{\rm{\mu }}}}}}$g m^−3^ 24-hour mean)^9^ (Supplementary Fig. 14). Ambient PM_2.5_ exposure due to crop residue burning is specifically associated with a three-fold greater risk of acute respiratory infection in the general Indian population^10^. Recent studies at local, urban and regional scales have shown that PM_2.5_ emitted from crop residue burning affects air quality not only in India but also across South Asia, including Pakistan, Nepal and Bangladesh, due to the transport by the predominantly northwesterly winds^7,9,11^.

Current regulations by the Indian government intended to reduce agricultural fires, including crop residue management, burning bans, and fines, have had limited efficacy^12,13^. Unlike other crop residue, the low protein content (e.g. N, P, K) and poor digestibility (e.g. high silicone and ash) of rice and wheat residue have limited their potential for use in biofuel, animal fodder, fertilizer and paper production (Fig. 1b, Supplementary Data 1). In addition, the tight schedule of the harvest-to-sowing transition under the predominant rice-wheat rotation cropping system in northwestern India have limited the rate of adoption of alternatives^12,13^. Crop residue burning allows cheap and fast disposal of crop residue and therefore remains a recurring issue, as revealed by a ~60% increase in the number of agricultural fires detected by NASA’s Aqua satellite from 2002 to 2016^9,14^.

Studies which can attribute air quality impacts to specific burning instances therefore help to inform targeted mitigation strategies and optimize resources for effective action on burning with minimal disruption to farmers^15^. Substantial work has been done on air pollution from fire emissions in individual, heavily polluted locations such as Delhi^7,9,11,14,16^. However, no work to date has related burning in each individual district to the eventual premature mortality risk and associated cost across India, where a large population experiences increased levels of pollution from fires. In addition, efforts to identify alternatives to burning have typically been qualitative or focused on national or regional measures such as adopting mechanized approaches and alternative crops^12,17,18^. This neglects the possibility that targeted changes in the timing and location of residue burning may be able to yield significant improvements, and that there might be large differences in the downwind health impacts resulting from the same amount of residue burning from specific locations. Such information is needed to support decision-making which can place public benefits such as health costs alongside the potential private costs to farmers^13^.

Here we aim to inform efforts to mitigate the adverse impacts of crop residue burning by quantifying how small-scale and targeted changes could affect the air pollution and health risks of the entire Indian population. We use the Global Fire Emissions Database v4.1s (GFEDv4.1s) to provide an estimate of emissions from open burning of crop residue, combined with district-level crop production data for India, to obtain a comprehensive map of the time and location of crop production and residue burning. We then use a regional atmospheric chemistry and transport model (GEOS-Chem adjoint) to perform inverse (receptor-oriented) simulations, computing the sensitivity of a given population’s exposure to PM_2.5_ with respect to emissions in any location at any time, and date. In combination with an Integrated Exposure Response (IER) function^3^ and an India-specific Value of Statistical Life (VSL)^19^, we use this data to estimate which burning events, in what locations, and at what times are responsible for the greatest increases in population exposure, premature mortality, and monetary societal cost during the period 2003–2019.

## Results and discussion

We use an adjoint modeling approach (GEOS-Chem adjoint) as our primary tool for air quality impact attribution. We first perform three sets of adjoint runs from which we obtain sensitivities that quantify the effect of emissions on population exposure for the whole Indian population (cost function $J$). Each set of runs represents one typical rainfall condition for a year (i.e. “flood” (2007), “drought” (2009), or “normal” (2012) year), but all three use the same population distribution. We combine this sensitivity data with fire emissions for each year from 2003 to 2019 to estimate the impact of agricultural residue burning on India-wide population exposure. Modifying the emissions dataset allows us to quantify the benefit of different targeted mitigation strategies, while using each of the three sensitivity datasets allows us to quantify the role of meteorological variability. For each year from 2003 to 2019, one of the three sensitivity datasets is chosen based on monsoon rainfall record in India (see Methods).

Two additional sets of adjoint simulations are performed for the “normal” meteorological year, in which the cost function $J$ is modified to include only either urban or highly populated areas. Comparison of results using these datasets to those using the full India-wide population allows us to evaluate the distribution of impacts between urban and rural areas.

For additional context, we perform 23 pairs of forward simulations with the conventional GEOS-Chem Classic model. Each pair simulates the post-monsoon burning season with and without India agricultural emissions, and the 23 pairs collectively cover the period from 1997 to 2019. This allows us to evaluate the impacts of Indian residue burning on surrounding countries, and to gain a more complete understanding of the interaction between population growth, meteorological variability, and historical changes in fire emissions. See Methods for a detailed description.

### Air quality impacts resulting from agricultural burning

**Fig. 2:**
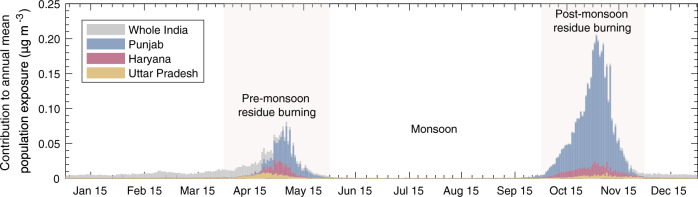
India-wide annual mean population-weighted PM2.5 exposure due to crop residue burning. Contributions are broken out for Uttar Pradesh (yellow bars), Haryana (red bars), Punjab (blue bars) and the rest of India (grey bars) by date, averaged over 2003–2019. The shaded area (light rose) in April-May and October–November denotes the dominant contribution from pre-monsoon fire season (wheat residue burning) and post-monsoon fire season (rice residue burning), respectively.

We define air quality impacts due to crop residue burning in terms of the premature deaths attributable to PM_2.5_ exposure and the associated monetized cost. Figure 2 shows how crop residue burning on each day contributed to the national mean PM_2.5_ exposure. From 2003 to 2019 we estimate that the annual mean population-weighted PM_2.5_ exposure due to burning activities in India averaged 6.7 ${{{{{\rm{\mu }}}}}}$g m^−3^. Pre-monsoon and post-monsoon residue burning contribute 28% and 64% of this total, respectively. Geographically, more than 90% of the India-wide fire-related exposure increase is due to agricultural fire emissions from the northwest states, with 64% from Punjab, 11% from Haryana, and 5.7% from Uttar Pradesh. It is consistent throughout 17 years that the pre-monsoon and post-monsoon fire seasons are responsible for 90% of PM_2.5_ exposure from residue burning, and that Punjab, Haryana, and Uttar Pradesh together contribute over two thirds of the nation-wide exposure burden (see Supplementary Data 2 and 3 for a breakdown by state and season). Demographically, urban and densely populated areas, defined by regions with a population density above 400 and 1,000 people per km^2^, respectively, are exposed to 2.0 ${{{{{\rm{\mu }}}}}}$g m^−3^ (31%) and 4.8 ${{{{{\rm{\mu }}}}}}$g m^−3^ (73%) greater annual PM_2.5_ concentrations than the national average due to residue burning (Supplementary Figs. 1, 31). This is supported by observations of elevated PM_2.5_ levels during burning seasons in large cities downwind of agricultural fires^12,14,16,17,20–22^.

By applying the IER and India-specific population distribution, we estimate 69,000 (95% CI: 57,000–80,000) total premature mortalities on average across India resulting from ambient PM_2.5_ exposure due to crop residue burning (Supplementary Data 4). Our estimate for 2015 (60,000, 95% CI: 50,000–70,000) is consistent with Global Burden of Disease (GBD) 2018 India Special Report’s estimate of premature mortality (66,000, 95 CI%: 57,000–79,000) attributable to agricultural fires in India for the same year^23^. Our estimate for the same period is 4.7–14% of premature mortality attributable to total ambient PM_2.5_ presented by earlier studies covering 2010-2019^4–6,15,23–28^. This fraction is consistent with findings from GBD 2018, where 6.1% of premature deaths in India due to ambient PM_2.5_ are attributed to agricultural fires^23^. The central estimates of early deaths as well as confidence intervals (CI) depend on the choice of relative risk function^29^. While the IER the has been applied worldwide, including India, to quantify attributable deaths due to biomass burning episodes^3,23–31^, we compare the IER-based results to those using other methods in the Methods and Supplemental Information.

Using a VSL adjusted for India, we estimate the annual, monetized cost of premature mortality due to crop residue burning as 23 (95% CI: 3.5–53) billion USD. This is equivalent to 38% of the total health expenditure, or 7.8% of the gross value added from agricultural activity on average (Supplementary Data 4). These two ratios have increased from 29% to 40%, and from 6.1% to 9.2%, respectively between 2003 and 2019 (Supplementary Data 4). We find that for any year in this period, the three largest contributors to these impacts are consistently Punjab (48–75%), Haryana (7.8–14%), and Uttar Pradesh (3.7–9.5%) (Supplementary Data 2-4).

### Contributions by district

**Fig. 3:**
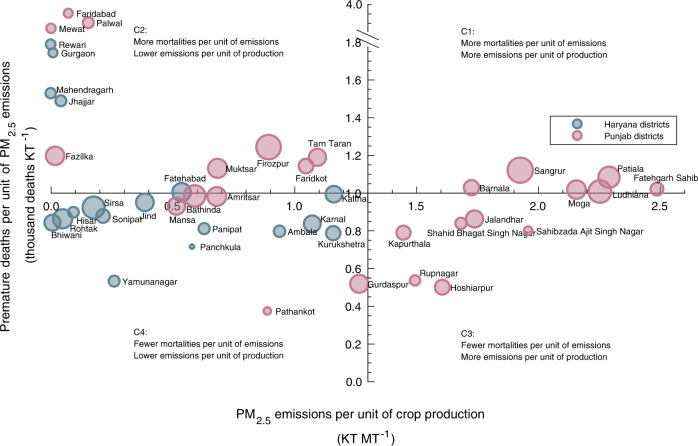
Premature mortalities due to PM2.5 exposure resulting from burning in districts of Punjab (blue) and Haryana (red). Districts are categorized by agricultural PM_2.5_ emissions per unit of crop production (kilo tonnes per million metric ton, or KT MT^−1^) on the x-axis, and premature deaths per unit of agricultural PM_2.5_ emissions (thousands KT^−1^) in y-axis, averaged over 2003–2019. The size (radius) of the dots indicates the gross crop production in MT of each district. The contribution to India-wide premature deaths from residue burning in each district is the product of emissions per unit of crop production (*x* axis value), mortalities per unit of emissions (y-axis value) and crop production (size of the dot) (Supplementary Figs. 2, 3).

Figure 3 shows the premature deaths per unit of emissions from burning (vertical axis) against the emissions per unit of crop production (horizontal axis) for each district in Punjab and Haryana. The contribution to premature mortalities per crop produced for these two states is larger than for the rest of India combined (Supplementary Figs. 2, 3). Variations between these districts are due not only to different crop production quantities but also different meteorology, population distributions, and agricultural practices. For the 43 districts in the two states, we use the two dimensions (emissions per unit production and mortalities per unit emission) shown in Fig. 3 to define four categories (C1–C4). Punjab and Haryana, under the rice-wheat rotation system, are collectively responsible for 60% of all rice and 30% of all wheat entering the central grain pool in India^12^. This results in a large amount of residue being burned per unit of crop production, as these two crops produce relatively high quantities of residue per unit of product compared to crops such as oilseed and sugarcane (Fig. 1b).

The contribution of crop residue burning in each district to total premature mortality in India is a product of three factors: first, premature deaths per unit of agricultural burning emissions (meteorology and population); second, agricultural burning emissions per unit of crop production (choice of crops/varieties); and third, the total crop production (Fig. 3). Districts in C1 rank high in the first two factors, partly because they grow coarse varieties of rice that generate more residue to be burned for the same amount of crop production^32^ and are mostly upwind of densely populated regions. As a result, they are on average responsible for 40% (27,000 deaths, valued at 9.0 billion USD) of the total air quality impacts in India due to burning, with Patiala and Sangrur alone contributing 20%. Compared to C1, districts in C2 and C3 have lower emissions per unit of crop production (<1.3 KT MT^−1^) and fewer premature deaths per unit of emissions (<1000 premature deaths KT^−1^), respectively. The contribution to the total air quality impact of residue burning is therefore 11% and 14% for districts in C2 and C3, respectively. The remaining districts (C4) are minor contributors to the total impacts, among which Kaithal has the largest contribution (2.4%) due to its cultivation of coarse rice (lower emissions per unit of production), lower sensitivity (fewer premature deaths per unit of emissions) and lower overall crop production (Supplementary Fig. 3). All Haryana districts fall in C2 and C4, whereas districts in C1 and C3 are all from Punjab, distinguished by the first factor (x-axis). Although both states are large rice producers, Haryana mainly grows basmati rice, the residue of which is used as animal feed. Punjab instead mainly grows non-basmati rice, the residue of which is not fed to livestock because of its high silica content and which is thus more often burned in-field^18,32^.

### Sensitivity of air quality impacts to spatial and temporal changes in emissions

**Fig. 4:**
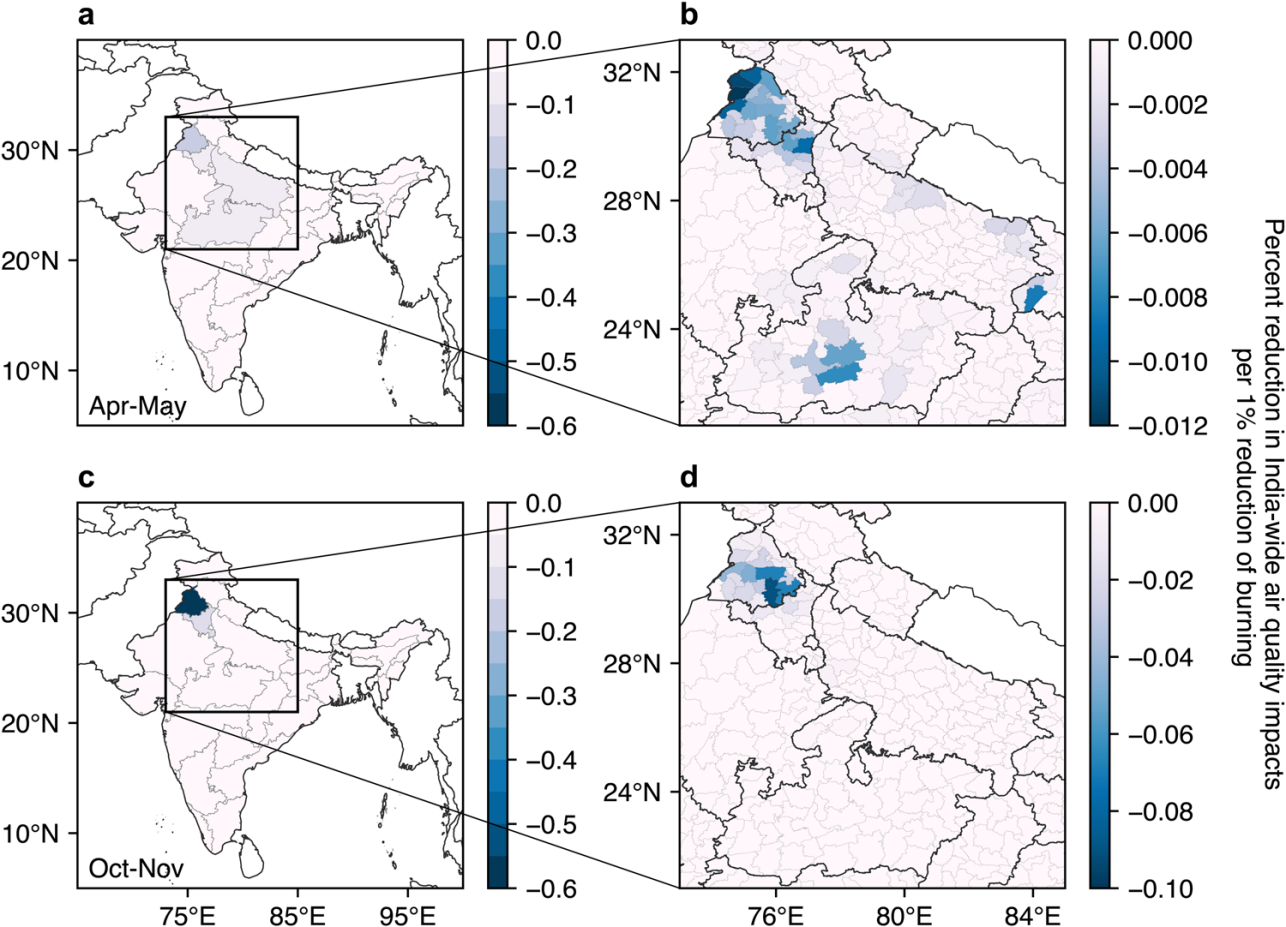
Sensitivity of India-wide particulate matter exposure to a reduction of burning in different locations. **a** Percent reduction in contribution to air quality impacts per 1% reduction in agricultural burning emissions in wheat residue burning season (April-May) in different states. **b** Zoom-in of a, i.e. districts in northwestern India. **c**, **d** are same as **a** and **b** but for rice residue burning season (October–November), hence the greater color bar limits as shown in d compared to b. India administrative maps are obtained from the Database of Global Administrative Areas, Version 4.1^50^. Coast outlines are plotted based on data in Python Matplotlib Basemap Toolkit, Version 1.2.1.

Figure 4 shows the percentage change in annual air quality impacts due to burning achieved by a 1% reduction in emissions from burning (hereafter the efficacy of reducing burning) in each state or district during two burning seasons averaged over 2003–2019. Specifically, during post-monsoon season, the efficacy of reducing burning is greatest in Punjab and Haryana, with a 0.57% and 0.065% reduction respectively in total India-wide impacts from crop residue burning per 1% reduction in burning emissions. This means 380 (95% CI: 320–450) and 45 (95% CI: 37–52) averted premature deaths, valued at 130 (95% CI: 20−300) and 15 (95% CI: 2.3−34) million USD, respectively, averaged across 17 years. Considering only burning emissions from Punjab, two thirds of the achievable benefit comes from reductions in burning in Sangrur, Patiala, and Ludhiana, where a 1% reduction in emissions from post-monsoon residue burning would result in a 0.18%, 0.05%, and 0.068% reduction, respectively, in India-wide burning-related air quality impacts.

One reason that farmers choose to burn agricultural residue rather than adopting (e.g.) mechanized alternatives is that there is a limited window of time, around two to three weeks, to manage crop residue between harvesting and sowing^9,13,16^. A water table preservation policy instituted in Punjab and Haryana in 2009 to align rice irrigation with the summer monsoon further shortened this window, which may have further intensified burning activities as farmers seek to ensure timely sowing for the next planting season^17,22^. This suggests that promoting burning earlier or later in the season may be unattractive or unachievable^10,13,17^.

**Fig. 5:**
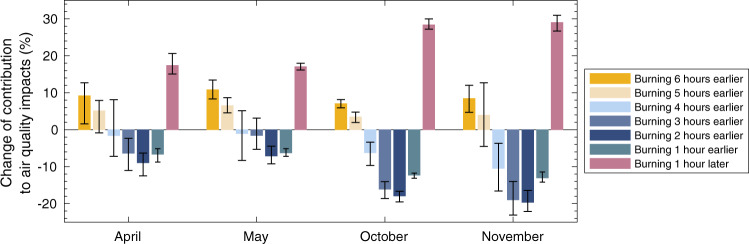
Change in air quality impacts resulting from shifts in the time of burning. Solid bars show the percentage change in contribution to annual residue burning-related air quality impacts in India achieved by shifting the burning peak from one hour earlier to six hours earlier, as well as one hour later, in different months, averaged over 2003–2019. The error bars depict the maximum and minimum percentage change for any single year in the period 2003–2019. The original peak of burning is set at 14:30 local time (LT) for both wheat and rice residue burning seasons. Diurnal cycles of emissions are based on MODIS satellite fire counts in the morning and afternoon (Supplementary Fig. 4).

It may instead be possible to burn earlier or later within a given day without incurring the same difficulties for farmers, but no study to date has quantified the potential benefits of such a change. We therefore quantify the air quality impacts of shifting burning within the day by applying different local time (LT) diurnal cycles of emissions (Supplementary Fig. 4). Figure 5 shows the changes in attributable air quality impacts resulting from shifts ranging from six hours earlier to one hour later.

Averaged over the period 2003–2019, burning earlier by one to four hours could reduce the total air quality impacts of crop residue burning by 0.5–19%, while burning too early or burning later could instead increase the impacts by 2−30%. For any individual year in that period, burning two to three hours earlier in November reduces the total, annual, residue burning-related contribution to India-wide air quality impacts by 15–23% (Fig. 5). If the target region is restricted to Punjab only, burning earlier by two hours in November yields an average (over 17 years) 14% reduction in air quality impacts resulting from that region’s residue burning (Supplementary Fig. 5). This means 9600 (95% CI: 8000–11,000) averted early deaths annually, valued at 3.2 (95% CI: 0.49–7.3) billion USD. This is greater than the sum from all other states if the same timing shift were applied. Although these improvements could be subject to the specific diurnal cycles we use, our assumptions of diurnal fire activity agree with previous findings that fires are typically set during early to mid-day (07:00–11:00 LT) and burn out by the evening (17:00–20:00 LT), lasting 13–15 hours^20–22,33^, and that fire activity generally peaks in the afternoon^22,32^. Liu et al. 2020 collected survey data from households in India, finding that despite regional variations, 97% of burning activities happen between 10:00 and 23:00 LT, nearly 30% of which happen in late evening (18:00–23:00 LT)^22^. While we do not have district-level information about hourly diurnal cycles of fire activities due to a lack of comprehensive in-field studies, our broad conclusion is that there may be significant benefits yielded by encouraging fires to be set earlier (10:00–13:00 LT) in the day rather than later (14:00–17:00 LT).

One contributing factor to these changes could be natural, diurnal changes in the depth of the planetary boundary layer (PBL). The PBL height (PBLH) typically peaks at 13:00–14:00 LT and decreases rapidly afterwards (Supplementary Fig. 6). It is a key meteorological parameter in pollutant dispersion^34–36^ because a higher PBLH favors dispersion and reduces aerosol accumulation^34,36^ (see Supplementary Information for a detailed discussion). However, the PBLH is also directly affected by aerosol loading meaning that the concentration of pollution can itself affect dispersion^35^. The PBLH decreases with increasing aerosol concentration, which enhances atmospheric stability and in turn favors even higher pollutant concentrations––a positive feedback loop^35^. However, the diurnal variations of PBLH do not change significantly on heavily polluted days compared to clean days^34,37^. Supplementary Fig. 6 also shows similar diurnal cycles of PBLH in polluted and less polluted areas. Therefore, despite local aerosol-PBL interactions, a shifted burning cycle on an hourly basis is not likely to significantly affect the broad patterns of PBLH and the timing effects of emissions. However, further investigation will be needed to evaluate how a shift in the diurnal pattern of burning (i.e. aerosol emissions) might in turn modify the diurnal pattern of PBLH––and therefore pollution dispersion––compared to the effects observed to date for existing emission patterns.

Other meteorological parameters including relative humidity (RH), temperature, and wind speed also vary diurnally and may affect the benefits of burning earlier^34,38^. Studies in India found higher pollutant concentrations with higher RH due to incomplete combustion, causing more secondary aerosol formation^38^. Supplementary Fig. 6 shows that, on average, RH is roughly constant from 11:30 to 15:30 local time. Our proposed shift moves the peak burning time from 14:30 to 13:30, suggesting that the RH effect is not significant. In addition, lower temperature and lower wind speed could trap more aerosols within the PBL^34,38^. This is consistent with our finding of decreased air quality benefits when the burning peak is too early or late (Fig. 5). Changes in wind speed due to high aerosol loading should also be considered in future online studies of specific interventions.

These findings suggest that aerosol-meteorology interactions are unlikely to eliminate the benefit of a shift in the diurnal pattern of burning. We recognize however that further studies involving sensitivity experiments will be needed to evaluate the degree to which these feedbacks suppress or enhance the benefit. In general, for any intervention our approach is a first-order tool, and more modeling, experiments and observations would be warranted before advancing any policy recommendation. However, our broad explanation is that the aerosols emitted when meteorology favors dispersion are more diluted and less harmful as they travel downwind (Supplementary Figs. 6, 30).

This work focuses on how India-wide air quality impacts are affected by any conceivable change in fire emissions using an “adjoint” modeling approach. Rather than tracking the transport of pollution and the distribution of air quality impacts for a single scenario, the adjoint model tracks the sensitivity of exposure over a disperse population with respect to changes in emissions sources. This is essential for efficiently evaluating different cost-effective pollution-abatement policies. We therefore do not calculate the local effects of shifting the timing of burning, e.g. the evolution of air pollution to downwind areas such as New Delhi.

### Inter-annual variations in the air quality impacts of residue burning

To provide context to the sensitivity-based results above, we use forward modeling to simulate the post-monsoon residue burning season over a 23-year period (Supplementary Figs. 8, 9). We first quantify population exposure to BC and OC (carbonaceous PM_2.5_) from all sources. For these simulations we include changes in residue burning emissions, population (total), and meteorology (see Methods). We find that, while the daily-mean population exposure varies, the geographical distribution of exposure and meteorological context (wind fields) remain similar. From 1997 to 2019, the national average population-weighted PM_2.5_ exposure has increased from 54 ${{{{{\rm{\mu }}}}}}$g m^−3^ to 75 ${{{{{\rm{\mu }}}}}}$g m^−3^, possibly due to increasing crop production (Supplementary Figs. 7, 8). Northwest India (Punjab, Haryana, Uttar Pradesh, Delhi) is the most polluted region during post-monsoon burning season, and the daily-mean population-weighted PM_2.5_ exposure in these areas is consistently higher than the national average. For every year from 1997 to 2019, we estimate that Delhi’s air consistently had levels of (carbonaceous) PM_2.5_ exceeding 120 ${{{{{\rm{\mu }}}}}}$g m^−3^ when averaged over the two-month post-monsoon burning period.

We also perform 23 counterfactual simulations, covering the same period, in which emissions from residue burning in India are not included. While our sensitivity simulations show that our broad conclusions (e.g. contribution by state, season) are consistent throughout 17 years of differing fire emissions and three different meteorological conditions (Supplementary Data), our extended set of forward simulations show that the average air quality impacts attributable to agricultural fires is 2.4% lower in drought years (e.g. 2009, 2015) and 4.8% higher in flood years (e.g. 2007, 2019). For years with normal rainfall (e.g. 2012, 2018), close (−0.8%) to the 17-year average, we estimate the annual fire-related premature deaths at 68,000 (95% CI: 57,000–79,000), valued at 22 (95% CI: 3.2–50) billion USD. Specifically, crop residue burning in 2016 contributes to the largest enhancement of nationwide PM_2.5_ exposure (9.6 ${{{{{\rm{\mu }}}}}}$g m^−3^), resulting in the most premature deaths 98,000 (95% CI: 82,000-110,000). This is consistent with the large number of agricultural fires observed by the Moderate Resolution Imaging Spectroradiometer (MODIS) instrument in that year, and the unprecedented enhancement in PM_2.5_ levels observed in Delhi (648 ${{{{{\rm{\mu }}}}}}$g m^-3^)^16,38^.

These results only include the post-monsoon burning season, and rely on monthly emissions data. Using our sensitivity data with daily emissions data and assigning each year from 2003 to 2019 an appropriate meteorological condition (flood year, drought year, normal year), we can quantify the impacts of each full year’s residue burning. From 2003 to 2019, along with an increase of food grain production from 210 MT to 300 MT (Supplementary Fig. 7), the monetized cost associated with burning increases from 7.2 (95% CI:1.1–17) to 44 (95% CI:6.7–100) billion USD (Supplementary Data 4).

A point of interest is the impacts in 2018 and 2019. In 2018, a subsidy was introduced to encourage mechanization as an alternative to burning^13^. Despite this policy change, we find air quality impacts attributable to crop residue burning to be similar between 2018 and 2019 at 86,000 premature deaths each year. To understand this, we re-derived impacts for 2015-2019 using fixed meteorological data. Under these conditions, mortalities in 2018 and 2019 are 4.2% and 11% lower than the previous three-year average, but 15% and 6.9% greater than for 2017, respectively (Supplementary Data 9). For just Punjab, Haryana, and Uttar Pradesh, the average exposure resulting from burning (assuming fixed meteorology) in 2018 and 2019 was 16% lower than the 2015–2017 average, but still 18% greater than the 2017 value alone (Supplementary Data 9).

This does not necessarily imply that the initiative was unsuccessful. Prior work has suggested that overall post-monsoon residue burning was decreased, showing a 18% reduction in fire counts observed by the MODIS satellite in northwest India in 2019 compared to the previous year (Supplementary Data 10). The GFEDv4.1s dataset suggests that the total dry matter burned in Punjab, Haryana, and Uttar Pradesh in 2018 and 2019 was only 9 and 3% less than the 2015–2017 average (Supplementary Data 11), respectively––but this masks two compensating changes. Focusing on the post-monsoon period only, the total dry matter burned in the three states in 2018 and 2019 was 36% below the 2015–2017 average, and only 1% greater than the 2017 value. Dry matter burned during the pre-monsoon period, however, was 52% and 130% greater in 2018 and 2019 respectively than the 2015–2017 average (Supplementary Data 11). With fixed meteorology, pre-monsoon residue burning contributed 38% of the total premature mortality resulting from fire emissions in these three states in 2019, compared to 9% in 2015 (Supplementary Data 9).

Although more data are needed to determine the significance of this trend, our results suggest that health benefits due to a reduction in emissions during the post-monsoon period in 2017-2019 have been offset by an increase in emissions during the pre-monsoon period and that the effectiveness of interventions should be considered across both seasons.

### Impacts of Indian agricultural residue burning on neighboring countries

The IGP is home to millions of people residing in not only northern India but also Nepal, Bangladesh and Pakistan. Agricultural fires in India are not subject to political borders and may have air quality impacts on neighboring countries as well. Using data from 23 years of forward simulations we find that PM_2.5_ exposure in Bangladesh, Nepal and Pakistan resulting from Indian crop residue burning is 0.24–12% of that in India (Supplementary Figs. 9, 10). Of these countries, impacts are lower in Bangladesh and Nepal (1.3–2.2%), whereas Pakistan’s PM_2.5_ enhancement averages 6.9% of that in India. We show that over 23 years, annual mean total increase in population exposure due to Indian crop residue burning is 4.5 $\times {10}^{9}$ people$\bullet {{{{{\rm{\mu }}}}}}$g m^−3^ in India, 2.6 $\times {10}^{8}$ people$\bullet {{{{{\rm{\mu }}}}}}$g m^−3^ in Pakistan, 7.8 $\times {10}^{7}$ people$\bullet {{{{{\rm{\mu }}}}}}$g m^−3^ in Bangladesh and 4.2 $\times {10}^{7}$ people$\bullet {{{{{\rm{\mu }}}}}}$g m^−3^ in Nepal. Therefore, the majority (88–95%) of burning-related population exposure is experienced by the Indian population. This is partly due to large-scale meteorological winds that carry pollution into, rather than away from, India (as shown in Supplementary Figs. 8, 9) as well as the high population density in this region.

### Implications of targeted interventions

In India, the total amount of crop residue (dry matter) generation has increased from 80 MT in 1950–1951 to 520 MT in 2017-2018 (Economic Survey of India, 2020). In recent years, crop residue burning has contributed to levels of PM_2.5_ concentrations that are 15–45 times higher than the WHO safety guidelines in northern India^9,16,21^. Our results suggest that this burning has a monetized annual cost of 23 billion USD averaged from 2003 to 2019, which has grown by a factor of six over the same period. Existing governmental efforts, including the National Policy for Management of Crop Residues, the National Green Tribunal Act, and the Straw Management System, are in place to reduce the practice of burning^12^. However, in-field burning remains prevalent, especially in the states of Punjab and Haryana^12–14,17^. The results of our work have several implications on mitigation strategies.

First, we find that under similar meteorology and rice-wheat cropping system, the attributable air quality impacts from Punjab are six times as much as those from Haryana, partly because Haryana districts mainly grow basmati rice, which is more utilizable than non-basmati rice cultivated in Punjab (Fig. 3)^18^. Therefore, in addition to diversifying rice with crops that produce less residue such as pulses and oilseeds (Fig. 1b), adopting rice varieties that are less burning-intensive could also reduce the amount of burned residue and eventual attributable fire-related impacts. Second, current regulations, including bans and fines, are mainly implemented at national or state levels, which may overlook the possibilities that local-scale actions can bring significant benefit. Our study shows that a small number of administrative areas may be prioritized for interventions to effectively reduce the attributable impacts from fires (Fig. 4), with burning in six districts in Punjab responsible for 40% of India-wide exposure to fire related PM_2.5_. Such information is helpful for spatially targeted decision-making. Third, we also find that burning earlier by a few hours within a day could avert up to 14% of the air quality impacts resulting from residue burning, over 90% of which is borne by Indians. Such temporally targeted interventions may therefore allow effective and potentially low-cost reductions in harm while local effects on pollution distribution can be further investigated. In addition, a combination of targeted decision-making and more permanent solutions, such as mechanization, may help to optimize resources and minimize disruption to farmers.

While this study implies that significant societal benefits can potentially be achieved by small-scale actions, a comprehensive cost-benefit analysis and consideration of extra incentives for farmers is needed for actionable planning and wide adoption of alternatives. Our hope is that work such as ours can provide quantitative data for near-term measures to effectively reduce the harms of agricultural residue burning while more holistic solutions can be pursued.

### Comparison with other approaches and studies

In the case of crop residue burning, most existing work focuses on air pollution at local and urban scales, highlighting the influence of agricultural fires on regional air quality^7,9–11,14,16,20,21^. However, impacts of fire emissions are likely to extend over a much larger area due to dispersion and transport^1,4,11,16^. Epidemiological studies show that any additional exposure to PM_2.5_ causes an increased mortality risk even when baseline exposure is very low^3,39^. This means that small exposure increases over large regions should be considered equally with focused increases over smaller regions^39^. Our study equitably evaluates the impacts on everyone who is subject to fire-related air pollution in India, not just those in a typical pollution hot-spot.

In addition, while severe air pollution has been observed in cities such as Delhi^7,9,16,22^, we acknowledge that crop residue burning is one of several factors in urban air pollution, rather than necessarily the dominant factor. For example, although agricultural emissions can contribute up to 50% of PM_2.5_ in Delhi during post-monsoon fire season, the dominant (70%) emission sources of year-round PM_2.5_ are vehicle, industrial and energy emissions^5,23^. For population in suburbs or rural areas, crop residue burning is a greater year-round contributor in absolute or relative terms. By using an adjoint modeling approach, our study relates the eventual impacts across the country back to burning in each hour and individual district, and shows that crop residue burning can be controlled independently to achieve the greatest reduction of aggregate exposure at the lowest cost.

The main contribution of this work is the quantification and disaggregation of air quality impacts across India due to agricultural emissions, and the identification of a potential new form of impact mitigation. However, our baseline estimates of attributable premature deaths in India also compare well with existing health impact assessment studies. GBD MAPS Working Group attributed 66,200 (6.1%) ambient PM_2.5_-related premature deaths to open burning of agricultural residue in 2015^23^, which is similar to our calculations for 2015 (60,000, 95% CI: 50,000–70,000). The total premature deaths attributable to ambient PM_2.5_ exposure in India range from 570,000 to 1,450,000 in previous studies covering years from 2010 and 2019^1,2,4–6,15,23–28^, with which our estimate is consistent (assuming a ~6% contribution from agriculture fires).

### Sources of uncertainty

Although we include an uncertainty analysis in the Supplementary Discussion (e.g. inventory uncertainty), our study is subject to several other sources of uncertainty that we do not quantify. First, we focus on premature mortality risk changes resulting from changes in exposure to primary PM_2.5_ released from residue burning and do not quantify exposure to other species such as ozone^29^. Second, the IER function used in this study assumes equal toxicity for all PM_2.5_ species and ignores differences in composition, which still requires further investigation^3^. We also recognize that the IERs, as with other commonly used concentration response functions, were not developed specifically for India. However, without comprehensive epidemiological studies and available established models for India, the IER function is still a practical solution to represent our best at-present understanding of relative risks attributable to PM_2.5_ exposure (see Methods). As Indian epidemiological evidence grows and concentration response function models are being developed and improved, future work may benefit from the adoption of a new method. Third, we assume a single diurnal cycle for burning emissions based on satellite information due to limited data of hourly burning activities from local sources (Supplementary Discussion, Supplementary Figs. 4, 28, 29). Lastly, since this study focuses on the broader air quality impacts over a large dispersion population, we do not specifically look at individual pollution hot-spots such as Delhi. We do however provide additional assessments for densely populated (urban) areas, where Delhi is a main recipient of pollution from agricultural fires (Supplementary Fig. 1).

### Future research

Our approach allows any proposed emissions change to be related to the eventual air quality impacts for the Indian population and sets the stage for future research into crop residue burning. Since we have focused most of our analysis on a single intervention, it would be a natural next step to examine the effects of such interventions in downwind locations (e.g. the spread of pollutants in New Delhi) using conventional forward modeling techniques. Online modeling considering aerosol-meteorology interactions (e.g. aerosol effects on PBL variations) is also needed to better understand whether these feedbacks would suppress or enhance reductions in exposure. Furthermore, since our assumed diurnal pattern of burning may not reflect true fire activities, focused observational work on burning practices is needed to verify that these benefits are realizable. In addition, a deep assessment (e.g. cost-benefit analysis) of any single alternative is needed to determine how plausible such an intervention would be in practice.

Our study estimates the total annual premature deaths and the value of mortality risk reduction attributable to PM_2.5_ exposure from crop residue burning in India over 2003–2019. We also estimate the efficacy of marginal changes to reduce these impacts at the district level, finding that a small number of administrative regions could be prioritized to provide the maximum air quality improvement. We find that six districts in Punjab are responsible for 40% of the nationwide air quality impacts as a result of meteorological factors, the size of the downwind population, and the use of residue-intensive crops. Our work provides additional insights into potentially low-cost interventions that may significantly reduce the air quality impacts, such as shifting to burning in the morning rather than afternoon and promoting less residue-intensive crops (e.g. basmati rice instead of non-basmati rice). These findings provide a quantitative basis for the design and optimization of mitigation strategies for crop residue burning on a broad scale, as well as providing new opportunities for future regional and local studies on agricultural fires in India.

## Methods

### Agricultural residue burning emissions

Consistent with GBD 2018 India Special Report, we calculate emissions from agricultural residue burning using the Global Fire Emissions Database v4.1s (GFEDv4.1s) from 2003 to 2019 (daily emissions for adjoint simulations) and from 1997 to 2019 (monthly emissions for forward simulations)^24,39^. GFEDv4.1s is a hybrid emissions inventory that incorporates satellite and ground-based measurements to estimate fire emissions of various types (including open burning of agricultural residue). In particular, it includes a small fire boost based on active fire detections outside the burned area extent, which improves estimation of emissions from frequent and/or short-lived burning events (e.g. agricultural fires)^40,41^. A comparison using alternative fire emissions inventories is provided in the Supplemental Information. Similar to Koplitz et al. 2016, we define burning-attributable PM_2.5_ as the sum of black carbon (BC) and organic carbon (OC), the primary components of fire smoke-related PM_2.5_^31^_._

The diurnal pattern of fire activity in the standard GFEDv4.1s product is estimated using an emissions redistribution approach. The diurnal cycles of burning are estimated based on observational data from geostationary satellites over the Americas, which are then applied to other parts of the world by matching three broad fuel types (crop/grass, shrub/savanna, and forest)^40,42^. While appropriate for many applications over North and South America, this method is not likely to accurately reflect agricultural residue burning in India because the crops grown, crop cycles, field size, and crop practices are different^40^. We therefore apply an alternative diurnal cycle for agricultural residue burning in India based on satellite information from prior literature. The fire activity in sub-tropical areas is typically more intense in the early- to late-afternoon^33,42^. Over India, the fire counts from MODIS Aqua (13:30 LT) are three to four times greater than those from Terra (10:30 LT) during periods of crop residue burning^14,17,22,42^. Based on this information, and in the absence of more reliable and/or accurate observational data specific to agricultural burning, we assume that agricultural burning emissions have a triangular profile (Supplementary Fig. 4), where 95% of emissions occur between 06:30 LT and 19:30 LT, with a peak at 14:30 LT. Sensitivity to this assumption is explored in Supplementary Discussion.

### Air quality modeling with the adjoint model

We use the adjoint (version 35) of the GEOS-Chem atmospheric chemistry and transport model to quantify the sensitivity of annual mean population exposure to PM_2.5_ in India with respect to emissions sources in the extended Asia domain (70 °E – 150 °E, 15 °S-55 °N)^43^. Adjoint simulations are performed at a resolution of 0.5° × 0.667° (latitude × longitude), with 47 uneven vertical layers from the surface up to 80 km altitude. Boundary conditions are saved from global runs at a resolution of 2° × 2.5°. The adjoint model quantifies the effect of changes in any emissions species (e.g. BC) at any time and any grid cell in India on a scalar quantity (cost function) $J$. In our case, the cost function is the India-wide population-weighted exposure to PM_2.5_. The adjoint approach has been widely applied in inverse (receptor-oriented) problems such as air quality impact attribution, which suits the need of this study^29,43^. We use GEOS-5 meteorological fields from the Goddard Earth Observing System of the NASA Global Modeling Assimilation Office and non-fire anthropogenic emissions from the Emissions Database for Global Atmospheric Research v4.3.2^44^. Each adjoint simulation first requires a conventional, forward simulation to be performed; the data from these forward simulations is compared against observational data in our model validation (below).

Two sets of simulations are run with the GEOS-Chem adjoint model. First, we perform three sets of simulations for three full years (2007, 2009, 2012) which respectively represent a typical rainfall condition for a “flood”, “drought” and “normal” year, based on 20-year monsoon rainfall data in India (Supplementary Fig. 7). Each set includes an adjoint run and a forward run (necessary for adjoint simulations). For each year we calculate the sensitivity of annual population-weighted exposure to PM_2.5_ across all of India (cost function $J$) with respect to emissions from December 1^st^ the previous year to January 31^st^ the year after. The first and the last month are discarded due to model spin-up and down, such that data for the whole year are used in the analysis. We then classify 2003–2019, where daily fire emissions are available, into three categories by meteorology type (Supplementary Fig. 7). By applying adjoint sensitivities with gridded agricultural fire emissions corresponding to their rainfall (meteorology) condition, we estimate the total change in population-weighted exposure for the entire Indian population due to emissions from crop residue burning for each year.

Second, we perform two other full-year adjoint simulations, where the cost function is modified to annual population-weighted PM_2.5_ exposure for population in urban areas and highly populated areas, for a typical “normal” year (Supplementary Fig. 1). We define urban and densely populated areas as locations in which the population density exceeds 400 and 1,000 people per km^2^, respectively. Besides estimating impacts on India as a whole, this allows us to separately quantify the impact of residue burning on different population groups, as people living in densely populated areas (e.g. IGP) may be exposed to different exposure levels than those living in rural areas (e.g. southern India).

### Air quality modeling with the forward model

To inform estimates of long-term trends in exposure and the spatial distribution of impacts, we use the “forward” model GEOS-Chem Classic (version 13.0.2) and perform 23 sets of conventional, forward-running simulations for September 1^st^ to December 31^st^ for each year between 1997 and 2019, where monthly fire emissions are available. September is discarded due to model spin-up, and only October to November are considered for the “post-monsoon season”. Each simulation is performed over the extended Asia domain (60° E-150 °E, 15 °S-55 °N) at a resolution of 0.5° × 0.625° (latitude × longitude), with 73 uneven vertical layers from the surface up to 80 km altitude. Similar to adjoint simulations, boundary conditions are saved from global runs at a resolution of 2° × 2.5°. Each set includes two simulations with and without Indian agricultural residue burning emissions, which provides information on the long-term impact of Indian post-monsoon crop residue burning on population living in neighboring countries including Bangladesh, Nepal and Pakistan. We use meteorological data from the Modern-Era Retrospective analysis for Research and Applications, Version 2 (MERRA-2) and monthly (rather than daily) agricultural residue burning emissions data from GFEDv4.1s. This data is also used in our model validation.

### Calculation of population exposure and source contribution

The adjoint sensitivities are partial derivatives of a cost function (PM_2.5_ exposure) with respect to various control parameters (emissions). Here the cost function for the adjoint simulation, $J$, is the annual mean population-weighted exposure to PM_2.5_ within India, including 29 states and seven union territories which are further divided into 666 administrative districts. The cost function ***j*** is defined as

1 $$J=\,\mathop{\sum }\limits_{i=1}^{{N}_{{{{{{\rm{lon}}}}}}}}\mathop{\sum }\limits_{j=1}^{{N}_{{{{{{\rm{lat}}}}}}}}\mathop{\sum }\limits_{t=1}^{T}\left[{\rho }_{{ij}}\bullet {\chi }_{{ijt}}\right]$$

where $i$ and $j$ are indices for the longitude and latitude, respectively; ${N}_{{{{{{\rm{lon}}}}}}}$ and ${N}_{{{{{{\rm{lat}}}}}}}$ are the number of grid cells in the longitudinal and latitudinal directions; $t$ is the time step; $T$ is the number of time steps in the simulation; ${\chi }_{{ijt}}$ is the surface-level PM_2.5_ in ${{{{{\rm{\mu }}}}}}$g m^-3^ at time step $t$ in grid cell ($i,j$); and ${\rho }_{{ij}}$ is the number of people in grid cell ($i,j$). Gridded population data are taken from the Global Rural Urban Mapping Project Gridded Population of the World v4 for 2020, available at https://sedac.ciesin.columbia.edu/data/set/gpw-v4-population-count-rev11. We do not vary the population distribution between years as this would not be a relevant control variable for emissions impact reduction, such that the estimate of exposure depends only on variations in the emissions inventory and in the meteorology data (i.e. the sensitivity data).

The three-dimensional sensitivity matrices we obtain from the adjoint at every time step $t$ are of the form

2 $${S}_{{ijt}}=\,\frac{\partial J}{\partial {E}_{{ijt}}}$$

where $J$ is the previously defined cost function, and ${E}_{{ijt}}$ is the emission (in kg per time step) of a species in the three-dimensional grid at time step $t$. *S*_*ijt*_ is therefore the change in annual-average population exposure to PM_2.5_ that results from a single additional kilogram of the given species at location *(I,j)* at time *t*.

We calculate the impact of crop residue burning on the change of annual population PM_2.5_ exposure in India as the summation

3 $$\Delta {P}_{{ijt}}=\,{S}_{{ijt}}\circ {E}_{{ijt}}$$

where the operator “$\circ$” is the inner product sign. $\Delta {P}_{{ijt}}$ is the change in annual-average PM_2.5_ exposure across all of India (units of person · ${{{{{\rm{\mu }}}}}}$g m^−3^) due to agricultural burning emissions ${E}_{{ijt}}$ (in kg) at location ($i,j$) and time $t$.

The adjoint method quantifies a linearized relationship between emissions and PM_2.5_ exposure. This makes it well suited for computing the impact of marginal emissions changes of a particular type at a particular location or time. Although there may be non-linearities that are not captured by this approach, the atmospheric processes relevant to PM_2.5_ (BC + OC)––wet deposition and advection––are accurately represented as linear operations in the GEOS-Chem model. As such, the error due to atmospheric non-linearities is expected to be small^29^.

Atmospheric chemistry-transport models depend on emissions inventories to compute air quality impacts, and our estimate and attribution of population exposure is subject to the specific choice of emissions inventory. While various global fire emissions inventories have been developed, differences across inventories such as satellite image interpretation and adjustment for small fires can result in large regional differences in emissions estimates^40^. We select six global emissions inventories, including five commonly used (e.g. Fire Inventory from the National Center for Atmospheric Research, Quick Fire Emissions Dataset) and one newly-developed for Indian agricultural residue burning, and make an inter-comparison by calculating PM_2.5_ exposure due to post-monsoon crop residue burning using each of the emissions inventories (Supplementary Discussion). We find that exposure estimates vary by up to a factor of seven due to uncertainty in emissions inventories (Supplementary Figs. 15–18). However, we find that this does not significantly affect our conclusions, which are focused on the relative reduction in harm which could be achieved through targeted interventions. Detailed comparison and discussion can be found in Supplementary Discussion.

### Model validation

To validate the model’s performance in simulating the impacts of residue burning, we compare estimated PM_2.5_ concentrations and aerosol optical depth (AOD) from the model output generated in the course of against satellite-and ground-based measurements in India. For surface PM_2.5_ we compare to annual estimates from the NASA Socioeconomic Data and Applications Center^45,46^. For AOD, we compare to estimates based on MODIS instruments on NASA’s Terra and Aqua satellites (https://ladsweb.modaps.eosdis.nasa.gov/archive/allData/61/), respectively. We also compare model-simulated AOD with observations from three available Aerosol Robotic Network (AERONET) sites in India (https://aeronet.gsfc.nasa.gov/cgi-bin/draw_map_display_aod_v3). In addition, we compare model outputs from the extended 23-year set of GEOS-Chem forward simulations against ground observations from India Central Pollution Control Board (https://app.cpcbccr.com/ccr/#/caaqm-dashboard-all/caaqm-landing/caaqm-data-availability) and US Embassy Air quality Monitors in India (https://www.airnow.gov/international/us-embassies-and-consulates/#India$New_Delhi) for recent years, where complete record of PM_2.5_ observations are available.

**Fig. 6:**
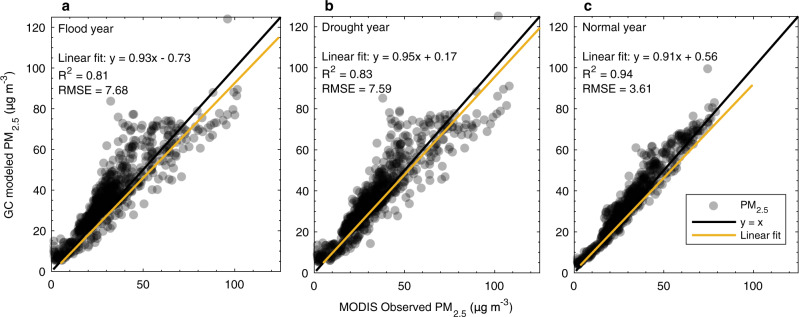
Model validation against satellite observation of PM2.5. **a** Comparison between ground-level PM_2.5_ from GEOS-Chem forward simulation which informed the adjoint runs and NASA MODIS satellite-derived observations for a drought year. **b**, **c** are same as a. but for flood and normal rainfall years, respectively. The black lines depict y = x, the yellow lines are linear regression using a least-squares fit between all available model and satellite PM_2.5_ values. Coefficients of linear fitting and root mean squared error (RMSE) are depicted at top-left of each sub-figure.

Figure 6 and Supplementary Fig. 11 show the comparison between simulated and satellite-derived annual mean ground-level PM_2.5_ concentrations over India. The GEOS-Chem forward model captures the broad spatial pattern and magnitude of annual mean ground-level PM_2.5_ across India. We find a positive bias of 4.3 ${{{{{\rm{\mu }}}}}}$g m^−3^ for flood, 1.8 ${{{{{\rm{\mu }}}}}}$g m^−3^ for drought, 2.0 ${{{{{\rm{\mu }}}}}}$g m^−3^ for normal years when averaged over all India, respectively. The model also captures high values (80-120 ${{{{{\rm{\mu }}}}}}$g m^−3^) over IGP from New Delhi eastward, with a mean bias from −0.53 to 2.6 ${{{{{\rm{\mu }}}}}}$g m^−3^ compared to satellite data. The model performs best in the normal year (*R* = 0.97) as compared to flood (*R* = 0.90) drought (*R* = 0.91) years. Disagreement is greatest in northeastern and eastern states (Supplementary Data 5). Compared to satellite-derived data, the model underestimates PM_2.5_ by 4.0–10 ${{{{{\rm{\mu }}}}}}$g m^−3^ in northwestern Uttar Pradesh, Sikkim, and Tripura, overestimates by 5.0–16.7 ${{{{{\rm{\mu }}}}}}$g m^−3^ in West Bengal, Bihar and Jharkhand, and overestimates by 4.6–9.7 ${{{{{\rm{\mu }}}}}}$g m^_3^ in Odisha and Chhattisgarh. The bias between simulated and satellite estimate is smaller in relative terms in southwestern and southern states (e.g. Maharashtra, Karnataka, Tami Nadu), with a mean overestimation at 1.7–4.6 ${{{{{\rm{\mu }}}}}}$g m^−3^. Further discussion about the causes of these discrepancies is included in Supplementary Discussion.

We also compare the model output to satellite AOD averaged between Level 3 MODIS/Aqua (13:30 LT) and MODIS/Terra (10:30 LT) on a monthly basis (Supplementary Fig. 12), since agricultural residue burning mostly occurs over the April-May and October–November periods. Prior to averaging, GEOS-Chem’s data is sampled at the same times as the valid observations from MODIS to ensure a consistent comparison. The simulated AOD shows overall seasonal variations consistent with MODIS over most regions in India when satellite data are available (January–June, October–December). In central and southern India (Maharashtra, Odisha, and Andhra Pradesh), GEOS-Chem has a positive bias of ~0.04–0.2 compared to MODIS estimates (~0.4–0.6). In northern India, the model has a negative bias of ~0.05–0.1 compared to MODIS (~0.7–0.9), especially in winter months (December, January and February). These biases are smaller (~±0.1) in March–April and October–November, showing a better model performance when agricultural burning occurs.

Simulated daily AOD is also compared with three AERONET sites located in New Delhi, Kanpur and Gandhi College (Supplementary Fig. 13). Accounting for available data points, model AOD correlates well with ground-based measurement (0.76$\le$ *R* $\le$ 0.82, 0.20 $\le$ RMSE $\le$ 0.30), with a mean difference ranging from −0.21 to −0.07. During wheat residue and rice residue burning seasons, the difference between model and AERONET is smaller by 0.02. Model performance in New Delhi (northwest India) shows a larger mean bias of -0.21, relative to a mean observed value of 0.81. Potential reasons for these differences are discussed in Supplementary Information.

The Central Control Pollution Board in India provides hourly ground observations of PM_2.5_ at 197 stations throughout India, including 37 stations in Delhi. We compare results from our extended set of forward simulations to ground observations from Central Control Pollution Board for 2018 and 2019 after performing data quality control procedures as detailed in Supplementary Discussion). Compared to ground-observations, our simulations capture the temporal variations and magnitude of local PM_2.5_ in Delhi in the post-monsoon season for both years, but have a mean negative bias of 6.9 ${{{{{\rm{\mu }}}}}}$g m^−3^. Our simulation also underestimates daily PM_2.5_ by as much as half on peak days (e.g. Nov 8^th^ 2018 and Nov 12^th^ 2019) during the post-monsoon residue burning period (Supplementary Fig. 14). However, our simulation considered only primary, carbonaceous PM_2.5_ (BC and OC). The peaks during this period are likely to include other/secondary PM_2.5_ components (e.g. sulfate aerosol) from non-fire sources which are not captured by our simulation. We make model-observation comparison and provide statistical metrics to evaluate city-level model performance where observed PM_2.5_ data are available (Supplementary Fig. 15), finding a reasonable correlation (*R* ~0.4–0.8) at 82% locations, including Delhi, Patiala and Chandigarh (Punjab), Kaithal and Faridabad (Haryana) (Supplementary Fig. 15, Supplementary Data 6). Despite the discrepancies at a few cities (e.g. *R* ~0.3 in Panchkula, Punjab), we focus on the average increased air quality impacts across India due to burning. Therefore, local biases are unlikely to significantly impact our estimates for population exposure and subsequent health impacts.

Finally, we verify that the forward and adjoint simulations are consistent by using both models to estimate population-weighted PM_2.5_ exposure under different burning scenarios (Supplementary Fig. 16, Supplementary Data 7). Details are provided in Supplementary Notes, but overall the inconsistency between forward and adjoint modeling in estimated exposure is less than 10% for all tests (Supplementary Data 7).

Overall, the GEOS-Chem model performs reasonably well in capturing the broad distribution, magnitude and variability of PM_2.5_ and AOD in India comparing with observations from various sources (shown and quantified in Fig. 6, Supplementary Figs. 11−15, 20, and Supplementary Data 6). This provides a reasonable and quantitative evaluation of the model’s accuracy and the robustness of our findings

### Estimation of premature mortality

Consistent with GBD 2018 India Special Report^23^, we use the Integrated Exposure Response (IER) function to quantify mortality risk changes due to annual mean PM_2.5_ exposure attributable to agricultural residue burning^3,22^ (Supplementary Fig. 16). The IER was developed to estimate the relative risk of premature mortality (early deaths) from each cause of death over the entire global range of long-term exposure to PM_2.5_^29^. This study considers five causes of mortality, including chronic obstructive pulmonary disease, ischemic heart disease, lower respiratory infections, lung cancer, and cerebrovascular disease. The IER function is expressed as

4 $${{{{{{\rm{RR}}}}}}}_{h}({\chi }_{{{{{{\rm{base}}}}}}})=\left\{\begin{array}{cc}1+{\alpha }_{h}\times \{1-{e}^{-{\beta }_{h}{({\chi }_{{{{{{\rm{base}}}}}}}-{\chi }_{o})}^{{\delta }_{h}}}\} & ({\chi }_{{{{{{\rm{base}}}}}}} > \,{\chi }_{o})\\ 1 & ({\chi }_{{{{{{\rm{base}}}}}}}\le \,{\chi }_{o})\end{array}\right.$$

where ${{{{{{\rm{RR}}}}}}}_{h}$ is the relative risk for disease $h$ given some exposure change between the observed baseline PM_2.5_ level ${\chi }_{{{{{{\rm{base}}}}}}}$ and the theoretical minimum-risk PM_2.5_ concentration ${\chi }_{o}$ (range: 2.4–5.9 ${{{{{\rm{\mu }}}}}}$g m^−3^) at grid cell ($i,j$) and time ${t}$. We adopt values of four IER parameters ${\alpha }_{h}$,$\,{\beta }_{h}$,$\,{\delta }_{h}$, ${\chi }_{o}$ for each disease from GBD 2017 and conduct 1000 sets of Monte Carlo simulations to determine the mean and 95% uncertainty intervals of premature mortality. We also use India-specific population age distribution and baseline mortality rates data from GBD 2019 and the United Nations, Department of Economic and Social Affairs, Population Division to calculate attributable premature mortality with the consideration of varying relative risk of a disease, such as ischemic heart disease and cerebrovascular disease on different age groups (Supplementary Figs. 17−19). A detailed description for applying the IER to adjoint sensitivities is given in the Supplementary Notes.

As with cohort studies on air quality impacts assessment and attribution, our estimates of premature mortality depend on the choice of concentration-exposure function. If instead of the IER we assume a log-linear relationship between PM_2.5_ exposure and premature mortality, and apply a log-linear function for short-term exposure^47^, our estimated annual premature deaths are reduced by approximately 56%. Using the Global Exposure Mortality Model^3^ or meta-regression-Bayesian, regularized, trimmed splines^1^ for long-term exposure risks would increase the estimated mortality by up to 45% (Supplementary Figs. 25–26, Supplementary Discussion). This reflects uncertainty about the true size of attributable mortality.

Burnett and Cohen, 2020 evaluated different concentration-exposure functions and suggested that the IER remains a useful and preferred method^30^. For example, the log-linear model and the Global Exposure Mortality Model are sensitive to high levels of PM_2.5_, which is the case for India^3,30^. The IER has been continuously updated and improved since its introduction over a decade ago, and applied by both GBD investigators and WHO at different scales^1–3,30^. Although not specifically developed for India, the applicability of the IER in the Indian context has been supported by the Steering Committee on Air Pollution and Health Related Issues of the Indian Ministry of Health and Family Welfare^23^. We thus believe the IER represents our best at-present understanding of exposure-attributable mortality risks.

The estimate of premature mortality is also subject to how we apply the concentration-exposure function. When calculating changes in attributable deaths due to burning, we use a uniform country-level baseline mortality rate (BMR) specified by disease, age group and year for India adopted from GBD 2019^1^. Chowdury and Dey, 2016 suggested using a state-specific BMR, adjusted as a function of gross domestic product (GDP) in India^24^. Using a GDP-based proxy of BMR, we find a 10–12% increase in calculated early deaths (Supplementary Fig. 27, Supplementary Information). While we would ideally use a BMR that accounts for the socioeconomic heterogeneity in a country like India, the accuracy of a GDP-based proxy is not clear and risks introducing additional error. We therefore do not use this adjustment in our central analysis.

### Quantification of monetized cost

Finally, we multiply the number of estimated burning-related premature deaths with the VSL to monetize the impacts of air pollution. The VSL is widely used in the US and European countries to assess the benefits of prevented premature deaths^29,30^. As original country-scale studies on VSL in India are limited, we use a benefit transfer method to adjust VSL from the US to India^33^. We estimate the India-specific VSL for each year between 2003 and 2019 as:

5 $${V}_{{{{{{{\rm{IN}}}}}}}_{y}}=\,\left[{V}_{{{{{{{\rm{US}}}}}}}_{1990}}\,\times \,{\left(\frac{{G}_{{{{{{{\rm{US}}}}}}}_{y}}}{{G}_{{{{{{{\rm{US}}}}}}}_{1990}}}\right)}^{{\varepsilon }_{1}}\times \,\frac{{D}_{y}}{{D}_{1990}}\right]\times {R}_{y}^{{\varepsilon }_{2}}$$

where ${{{V}}}_{{{{{{{\rm{IN}}}}}}}_{{{{{{\rm{y}}}}}}}}$ is the VSL for India in year ${{{{{\rm{y}}}}}}$; ${{{V}}}_{{{{{{{\rm{US}}}}}}}_{1990}}$ is the VSL for the US in 1990, which is translated into VSL for the US in a target year $y$ (in square brackets); ${{{G}}}_{{{{{{\rm{U}}}}}}{{{{{{\rm{S}}}}}}}_{y}}$ is the real GDP per capita in the US for year $y$; ${\varepsilon}_{1}$ is the income elasticity for the US between different years; ${\varepsilon }_{2}$ is the income elasticity between the US and India; ${D}_{{{{{{\rm{y}}}}}}}$ is the GDP implicit price deflator for year $y$; and *R*_y_ is the income ratio of GDP per capita in purchasing power parity between India and the US in year $y$.

The US Environmental Protection Agency (EPA) provides an estimate for the VSL in the US in 1990 that follows a Weibull distribution (US EPA 2014). We use this as our base estimate of ${{{V}}}_{{{{{{{\rm{US}}}}}}}_{1990}}$, propagating the estimated uncertainty (in 95% CI) through all subsequent calculations using a Monte Carlo approach. The GDP per capita and GDP deflator in each year for the US is obtained from the Federal Reserve Bank of St Louis (https://fred.stlouisfed.org/series/A939RX0Q048SBEA#0). For the US-specific income elasticity ${\varepsilon }_{1}$ we assume a value of 0.7, based on estimates from the US EPA guideline. We also assume a value of 1.5 for the US-India income elasticity ${\varepsilon }_{2}$, based on previous research into countries with mean incomes substantially lower than the US^20,48,49^. *R* is calculated as the ratio of GDP per capita in purchasing power parity between India and the US, which we obtain from World Bank (https://data.worldbank.org/indicator/NY.GDP.PCAP.PP.KD).

Using this approach, we estimate a mean VSL of 0.16–0.51 million USD for India over 2003–2019. Specifically, the World Bank Group and the Institute for Health Metrics and Evaluation estimated a VSL of 0.6 million USD in 2013, which falls within the uncertainty range of our estimation for the same year (0.052–0.76 million USD). The VSL is an aggregation of individuals’ willingness to pay for a small reduction of their own mortality risk (e.g. 1 in 10,000 decreases in the chance of dying prematurely)^48^, therefore the monetary value of mortality risk reduction becomes the VSL value multiplied by the number of early deaths. The monetized cost of premature mortalities $\triangle {{{M}}}$ due to PM_2.5_ exposure from agricultural burning emissions in grid cell (${i},\ {j}$) at time $t$ is

6 $${C=V}_{{{{{{\rm{IN}}}}}}}\times \,\triangle M$$

### Uncertainty quantification

We use Monte–Carlo simulations to account for multiple sources of uncertainty. We perform 1,000 simulations, and in each simulation perform one random draw for each random variable. For the health response, we choose randomly from the 1,000 precalculated shapes of each IER function with ranges of four parameters. This means one draw and one function for each cause of premature death, as described previously. In each simulation, we also draw randomly from the Weibull distribution described by the EPA for the 1990 US VSL, which we assume to be the dominant uncertainty in calculation of the transferred VSL for India (Supplementary Data 4). Other sources of uncertainty, such as uncertainty in the satellite observations and agricultural fire emissions inventory, are not quantified in the central calculation but are investigated using inter-comparison and sensitivity analyses (Supplementary Figs. 21–24, Supplementary Discussion).

## Supplementary information

Supplementary Information

Description of Additional Supplementary Files

Supplementary Data 1

Supplementary Data 2

Supplementary Data 3

Supplementary Data 4

Supplementary Data 5

Supplementary Data 6

Supplementary Data 7

Supplementary Data 8

Supplementary Data 9

Supplementary Data 10

Supplementary Data 11

## Data Availability

The fire emissions data from GFEDv4.1s is available at the Global Fire Emissions Database (https://www.globalfiredata.org/data.html). India-specific IER curves by age group are available at http://ghdx.healthdata.org/record/ihme-data/gbd-2017-burden-risk-1990-2017. Gridded India population data is available at the Socioeconomic Data and Applications Center (https://sedac.ciesin.columbia.edu/data/set/gpw-v4-population-count-rev11). India-specific population distribution by age group is available at United Nations, Department of Economic and Social Affairs, Population Division (https://population.un.org/wpp/Download/Standard/Population/). India-specific baseline mortality rates are available at http://ghdx.healthdata.org/gbd-results-tool. Satellite-derived PM_2.5_ data is available at the Socioeconomic Data and Applications Center (https://sedac.ciesin.columbia.edu/data/set/sdei-global-annual-gwr-pm2-5-modis-misr-seawifs-aod-v4-gl-03). Observed PM_2.5_ at Indian ground stations is available at the Central Pollution Control Board of India (https://app.cpcbccr.com/ccr/#/caaqm-dashboard-all/caaqm-landing/caaqm-data-availability). Observed PM_2.5_ by US Embassy monitors in India is available at https://www.airnow.gov/international/us-embassies-and-consulates/#India$New_Delhi. The data for MODIS AOD is available at https://ladsweb.modaps.eosdis.nasa.gov/archive/allData/61/. The data for AERONET AOD in India is available at https://aeronet.gsfc.nasa.gov/cgi-bin/draw_map_display_aod_v3. India yearly, crop-wise and district-level crop production data and rainfall data are available at the Ministry of Statistics and Program Implementation, Ministry of Agriculture and Farmers Welfare (https://agricoop.nic.in/en), Indiastat (https://www.indiastat.com/data/agriculture) and Open Government Data Platform India (http://data.gov.in). The data for the US and India historical GDP is available at https://data.worldbank.org/indicator/NY.GDP.PCAP.PP.KD. All other processed data produced for analysis in this work can be found in Supplementary Discussion and Supplementary Data, or is available upon reasonable request to the first author (RL).

## Code availability

The code for the adjoint of GEOS-Chem atmospheric chemistry and transport model is available at http://adjoint.colorado.edu:8080/ (registration for Gitlab needed). The code for the GEOS-Chem forward model is available at 10.5281/zenodo.4681204.
