# Supplementary material for: Air quality impacts of crop residue burning in India and mitigation alternatives
Source: Nat Commun. 2022 Nov 14;13:6537. doi: 10.1038/s41467-022-34093-z (PMC9663555; doi:10.1038/s41467-022-34093-z)
Supplement: Supplementary file 1 — Supplementary Information [file 41467_2022_34093_MOESM1_ESM.pdf]

Supplementary Information: Air quality impacts from crop residue burning in India and mitigation alternatives

Ruoyu Lan[1,2], Sebastian D. Eastham[1,3], Tianjia Liu[4], Leslie K. Norford[2], and Steven R.H. Barrett[1,3]

[1]Laboratory for Aviation and the Environment, Massachusetts Institute of Technology, Cambridge, MA 02139, USA
[2]School of Architecture and Planning, Massachusetts Institute of Technology, Cambridge, MA 02139, USA
[3]MIT Joint Program on the Science and Policy of Global Change, Massachusetts Institute of Technology, Cambridge, MA 02139, USA
[4]Department of Earth and Planetary Sciences, Harvard University, Cambridge, MA 02138, USA

Corresponding author: S.D. Eastham (seastham@mit.edu)

Supplementary Notes

1. Consistency between forward and adjoint model

We estimate the changes of annual mean population-weighted exposure due to biomass burning emissions from forward simulations as 13.95 μg m^{-3}, in agreement with the GEOS-Chem Adjoint estimation of 15.12 μg m^{-3} (+8.39%). In addition, we perform multiple simulations with different emission scenarios (Supplementary Fig. 16) to compare agreement between the adjoint and forward simulation estimates of biomass burning impacts. Supplementary Data 7 lists the month-averaged population weighted exposure from six simulations covering Apr 15-May15 and Oct 15-Nov 15, 2009, representing two major burning seasons. The adjoint model overestimates $PM_{2.5}$ exposure by 0.29-2.29 μg m^{-3} (4.04-10.94%) compared to estimates from the forward model, and it shows a better agreement in post-monsoon season (6.11%) than in pre-monsoon season (9.03%). When emissions are masked partially in India, the $PM_{2.5}$ exposure estimated by the adjoint model shows a slightly larger bias (0.42-3.11%) (Supplementary Data 7).

2. Mortality estimation using IER

The IER function is expressed as:

$$\mathrm{RR}_h(\chi_{\mathrm{base}}) = \begin{cases} 1 + \alpha_h \times \left\{1 - e^{-\beta_h(\chi_{\mathrm{base}}-\chi_o)^{\delta_h}}\right\} & (\chi_{\mathrm{base}} > \chi_o) \\ 1 & (\chi_{\mathrm{base}} \leq \chi_o) \end{cases} \quad (1)$$

where RR_h is the relative risk for disease h given some exposure change between the observed baseline $PM_{2.5}$ level χ_{base} and the theoretical minimum-risk $PM_{2.5}$ level χ_o (range: 2.4–5.9 μg m^{-3}, consistent with GBD 2019) at grid cell (i, j) and time t. We adopt parameters for each disease α_h, β_h, δ_h and χ_o from GBD 2017 and conduct Monte-Carlo simulations to compute uncertainty intervals. Supplementary Data 8 shows the estimated mortality for each cause of death due to increased exposure attributed to agricultural burning as well as the uncertainties. A single value is calculated for each parameter (α_h, β_h, δ_h, χ_o) of COPD, LRI, and LC; 16 groups of age-specific values (at five-year intervals between age 25 and 95, 95+) are calculated for parameters of IHD and CEV. The RR_h curves based on IER for a given exposure to $PM_{2.5}$ of each disease are given in Supplementary Fig. 17. Comparing RR at different exposure levels allows an estimation of the likely change in incidence of a specific disease ΔI_h. The expected mortality change for a specific age group k in total population due to an increase in $PM_{2.5}$ ($\Delta\chi$) for disease h in grid cell (i, j) at time t is:

$$\Delta M_{h,k} = \rho_k \cdot \rho \cdot \Delta I_h = \rho_k \cdot \rho \cdot I_h(\chi_{\mathrm{base}}) \cdot \frac{\mathrm{RR}_h(\chi_{\mathrm{base}} + \Delta\chi) - \mathrm{RR}_h(\chi_{\mathrm{base}})}{\mathrm{RR}_h(\chi_{\mathrm{base}})} \quad (2)$$

where ρ_k is the fraction of population of a specific age category k (Supplementary Fig. 18), which we obtain from United Nations Department of Economic and Social Affairs, Population Division (https://population.un.org/wpp/Download/Standard/Population/) and ρ is the total population in grid cell (i, j), which is taken from the Global Rural Urban Mapping Project Gridded Population of the World v4 product, available at https://sedac.ciesin.columbia.edu/data/set/gpw-v4-population-count-rev11. $I_h(\chi_{\mathrm{base}})$ is the baseline incidence (mortality) rate for disease h at $PM_{2.5}$ level χ_{base} (Supplementary Fig. 19) estimated by Global Burden of Disease

(GBD 2019, available at http://ghdx.healthdata.org/record/ihme-data/gbd-2017-burden-risk-1990-2017).

The adjoint approach provides only the impact of a change in population exposure due to agricultural burning (ΔP in Section 2.2) in grid cell (i, j) at time t. To apply adjoint sensitivities to Supplementary Equation (2), we perform a linearization based on the rate of change of RR (slope k between $\mathrm{RR}_h(\chi_{\mathrm{base}} + \Delta\chi)$ and $\mathrm{RR}_h(\chi_{\mathrm{base}})$ with respect to χ (Supplementary Fig. 20).

Given the definition of RR:

$$\mathrm{RR}_h(\chi_{\mathrm{base}}) = \frac{I_h(\chi_{\mathrm{base}})}{I_0} \quad (3)$$

where $I_h(\chi_{\mathrm{base}})$ is the observed baseline incidence rate due to disease h at baseline $PM_{2.5}$ level χ_{base}, we use the satellite-based estimate of χ_{base} by Hammer et al. (2022) [1] for each year simulated, and I_0 is the counterfactual incidence rate under the theoretical minimum-risk $PM_{2.5}$ level χ_0 (range: 2.4–5.9 μg m^{-3}). In reality, I_0 is not observable; to calculate the difference between the incidence rate at χ_{base} with a perturbation $\Delta\chi$, which is $I_h(\chi_{\mathrm{base}} + \Delta\chi)$, and without perturbation $I_{\mathrm{h}}(\chi_{\mathrm{base}})$ without knowing I_0, we use:

$$I_h(\chi_{\mathrm{base}} + \Delta\chi) - I_h(\chi_{\mathrm{base}}) = I_h(\chi_{\mathrm{base}}) \cdot \frac{I_h(\chi_{\mathrm{base}} + \Delta\chi) - I_h(\chi_{\mathrm{base}})}{I_h(\chi_{\mathrm{base}})} \quad (4)$$

$$\Delta I_h = I_h(\chi_{\mathrm{base}}) \cdot \frac{\mathrm{RR}_h(\chi_{\mathrm{base}} + \Delta\chi) - \mathrm{RR}_h(\chi_{\mathrm{base}})}{\mathrm{RR}_h(\chi_{\mathrm{base}})} \quad (5)$$

$$\frac{\Delta I_h}{I_h(\chi_{\mathrm{base}})} = \frac{\mathrm{RR}_h(\chi_{\mathrm{base}} + \Delta\chi) - \mathrm{RR}_h(\chi_{\mathrm{base}})}{\mathrm{RR}_h(\chi_{\mathrm{base}})} \quad (6)$$

The RR is nearly linear between χ_{base} and $\chi_{\mathrm{base}} + \Delta\chi$; applying the Taylor series expansion to $\mathrm{RR}_h(\chi)$ around the point $\mathrm{RR}_h(\chi_{\mathrm{base}})$ and $\mathrm{RR}_h(\chi + \Delta\chi)$ also around $RR_h(\chi_{\mathrm{base}}))$ $(\chi \geq \chi_{\mathrm{base}})$, thus yields:

$$\mathrm{RR}_h(\chi) = \mathrm{RR}_h(\chi_{\mathrm{base}}) + (\chi - \chi_{\mathrm{base}})\left(\frac{\partial \mathrm{RR}_h}{\partial \chi}\right)_{\chi=\chi_{\mathrm{base}}} + \frac{(\chi - \chi_{\mathrm{base}})^2}{2!}\left(\frac{\partial^2 \mathrm{RR}_h}{\partial^2 \chi}\right)_{\chi=\chi_{\mathrm{base}}} + \cdots \quad (7)$$

$$\begin{aligned}\mathrm{RR}_h(\chi + \Delta\chi) = {} & \mathrm{RR}_h(\chi_{\mathrm{base}}) + (\chi + \Delta\chi - \chi_{\mathrm{base}})\left(\frac{\partial RR_h}{\partial \chi}\right)_{\chi=\chi_{\mathrm{base}}} \\ & + \frac{(\chi + \Delta\chi - \chi_{\mathrm{base}})^2}{2!}\left(\frac{\partial^2 \mathrm{RR}_h}{\partial^2 \chi}\right)_{\chi=\chi_{\mathrm{base}}} + \cdots\end{aligned} \quad (8)$$

where $\left(\frac{\partial \mathrm{RR}_h}{\partial \chi}\right)_{\chi=\chi_{\mathrm{base}}}$ is the gradient of the IER function at the observed baseline exposure level. With Supplementary Equations (7) and (8), a change of RR can be written as:

$$\mathrm{RR}_h(\chi_{\mathrm{base}} + \Delta\chi) - \mathrm{RR}_h(\chi_{\mathrm{base}}) = \Delta\chi \left(\frac{\partial \mathrm{RR}_h}{\partial \chi}\right)_{\chi=\chi_{\mathrm{base}}} + O(\Delta^2\chi) \tag{9}$$

To estimate the error of $O(\Delta^2\chi)$ in Supplementary Equation (9), for example, for drought years, apply χ_{base} = 74.0 μg m^{-3} [1] and $\chi_{\mathrm{base}} + \Delta\chi$ = 60.05 μg m^{-3} ($\Delta\chi = -13.95$ μg m^{-3} from GEOS-Chem forward simulations). For LRI (Supplementary Fig. 17), according to Supplementary Equation (5) $\mathrm{RR}_h(\chi_{\mathrm{base}} + \Delta\chi) - \mathrm{RR}_h(\chi_{\mathrm{base}}) = -0.18$, and according to Supplementary Equation (9), $\mathrm{RR}_h(\chi_{\mathrm{base}} + \Delta\chi) - \mathrm{RR}_h(\chi_{\mathrm{base}}) \sim -0.17$, thus $O(\Delta^2\chi) \sim 5.9\%$. From Supplementary Fig. 17, the RR curve for LRI has the largest slope between χ_{base} and $\chi_{\mathrm{base}} + \Delta\chi$ compared to LC, COPD, CEV and IHD; thus $O(\Delta^2\chi)$ is no larger than 6% for the approximation in Supplementary Equation (9), which allows impacts to be calculated using aggregated estimates of exposure for all diseases.

Then Supplementary Equation (6) becomes:

$$\frac{\Delta I_h}{I_h(\chi_{\mathrm{base}})} = \frac{\Delta\chi}{\mathrm{RR}_h(\chi_{\mathrm{base}})}\left(\frac{\partial \mathrm{RR}_h}{\partial \chi}\right)_{\chi=\chi_{\mathrm{base}}} \tag{10}$$

Applying Supplementary Equation (10) to Supplementary Equation (2):

$$\Delta M_{h,k} = \rho_k \cdot \rho \cdot I_h(\chi_{\mathrm{base}}) \cdot \frac{\Delta I_h}{I_h(\chi_{\mathrm{base}})} \tag{11}$$

$$\Delta M_{h,k} = \rho_k \cdot [\rho \cdot \Delta\chi] \cdot I_h(\chi_{\mathrm{base}}) \cdot \left[\frac{1}{\mathrm{RR}_h(\chi_{\mathrm{base}})} \left(\frac{\partial \mathrm{RR}_h}{\partial \chi}\right)_{\chi=\chi_{\mathrm{base}}}\right] \tag{12}$$

where $\rho \times \Delta\chi$ is the population exposure change due to emissions obtained from the GEOS-Chem adjoint simulations. To calculate the impact of an increase in exposure due to agricultural burning, we apply agricultural burning emissions E to adjoint sensitivities S to gain ΔP (equivalent to $\rho \times \Delta\chi$) as described in Section 2.2. Thus, Supplementary Equation (12) becomes:

$$\Delta M_{h,k} = \rho_k \cdot (S \circ E) \cdot I_h(\chi_{\mathrm{base}}) \cdot \left[\frac{1}{RR_h(\chi_{\mathrm{base}})} \left(\frac{\partial \mathrm{RR}_h}{\partial \chi}\right)_{\chi=\chi_{\mathrm{base}}}\right] \tag{13}$$

where "∘" is the inner product sign of the two matrices. The estimated burning-related mortality E over India is $\Delta M = \sum_{ijt} \sum_h \sum_k \Delta M_{h,k}$.

Using this method, the premature mortalities due to total fire emissions (e.g. forest fires, peat land fires, agricultural fires) based on GEOS-Chem forward and adjoint simulation results are estimated as 152,000 and 165,000, respectively.

Supplementary Discussion

1. Model uncertainties

1.1 Inventory uncertainty and inter-comparison

As stated in Methods in the main text, though the forward model performs reasonably well, there is still some disagreement between the forward GEOS-Chem simulation and MODIS in both $PM_{2.5}$ and AOD in parts of India.

There are several possible causes for these discrepancies. Considering first biomass burning emissions, the model does not account for plume rise, injecting them only into the atmospheric boundary layer. This is likely a source of error for regions with significant biomass burning (e.g. deforestation) and may partially explain the differences in distribution over the IGP, where forest fire events can have significant influence [2,3]. Our focus is agricultural fires, which are often set by small individuals (i.e farmers) and do not typically produce large buoyant plumes, and this particular weakness in the model is therefore not relevant to our evaluation even though it will cause an error in comparison to observations of overall $PM_{2.5}$.

A second source of error is the inaccuracy in the fire emissions inventories. Atmospheric chemistry and transport models depend on emissions inventories to compute air quality impacts. Many emissions inventories use satellite measurements (e.g. active fires, burned area, fire energy) to estimate total emissions and distribution. However, differences across inventories such as satellite image interpretation and adjustment for small fires can result in large regional differences in emissions estimates [4]. For example, the GFEDv4.1s fire emissions inventory has known inaccuracies due to limitations in the method used to estimate emissions from burned areas, as with other burned area-based emissions inventories [2,4,5]. To address (a) uncertainties regarding biomass burning emissions in emissions inventories estimated from satellite observations as well as (b) discrepancies between simulated and observed $PM_{2.5}$ due to (a), we focus on Indian post-monsoon agricultural residue burning and present an inter-comparison of model estimates of fire-related exposure to $PM_{2.5}$ using six global emissions inventories, including five widely-used emission inventories: (1) Global Fire Emissions Database (GFEDv4.1s, [6]), (2) Fire Inventory from NCAR (FINNv1.5, [7]), (3) Global Fire Assimilation System (GFASv1.2, [8]), (4) Quick Fire Emissions Dataset (QFEDv2.5r1, [9]), and (5) Fire Energetics and Emissions Research (FEERv1.0-G1.2, [10]) and one emissions inventory newly developed for Indian agricultural residue burning: Survey Constraints on FRP-based Agricultural Fire Emissions in the Indo-Gangetic Plain (SAGE-IGP, [11]). Specifically, SAGE-IGP addresses the uncertainties in Indian agricultural fire emissions (e.g. fuel consumption and the fire diurnal cycle) with a validation using burn rates from the household survey and government crop production statistics in India [11,12].

Among six fire emissions inventories, GFEDv4.1s, FINNv1.5, and SAGE-IGP, follow a bottom-up approach, which we broadly defined as aggregations of local data (e.g. burned area, fuel consumption), whereas GFASv1.2, QFEDv2.5r1 and FEERv1.0-G1.2 follow a top-down approach, which we define as segregations of total emissions using geographical parameters (e.g. fire radiative power, aerosol optical depth). Supplementary Fig. 21 shows a comparison of primary $PM_{2.5}$ (BC+OC) emissions in post-monsoon residue burning season (Oct 1st -Nov 30th) in India. GFEDv4.1s and GFASv1.2 are at the lower end (6.2-12.6 Gg/year) and FEERv1.0-G1.2 is at upper end (95-150 Gg/year) (Supplementary Fig. 21). Such disagreement in magnitude and temporal variability in India across emissions inventories is consistent with findings for IGP (Indo-Gangetic Plain) by Liu et al. 2020 [11].

There are five major sources of uncertainties in satellite observations that are used to construct emissions inventories: (1) active fires; (2) fires obscured by cloud/smoke; (3) fragmentation of burned area; (4) topography complexity; (5) undetectable small fires [4]. Due to different combinations of observations, cloud correction algorithms and assumptions of emissions factors, fire emissions estimate in India in emissions inventories differ by an order of magnitude. Choice of emissions inventory can significantly impact modeled results of air quality and health impacts, as found in the case of India post-monsoon residue burning [5].

As with Cusworth et al. (2018) and Koliptz et al. (2016) [5, 13], we assume the sum of BC and OC emissions from dry matter burned in the agricultural sector in October and November are agricultural residue burning emissions. We apply adjoint sensitivities to six emissions inventories to compute the variations of contribution to annual-mean population exposure due to fire emissions in post-monsoon season among emissions inventories (Supplementary Fig. 22). Contribution to exposure estimated from GFEDv4.1s and GFASv1.2 (5.7-7.5 μg m^{-3}) is ~12%-25% of that estimated from FEERv1.0-G1.2 (30-47 μg m^{-3}). Disagreement in population exposure estimates (as large as by a factor of 7.3) using different emissions inventories may propagate into the estimate of premature deaths and monetized costs due to crop residue burning.

There have been cohort studies identifying and quantifying regional discrepancies and uncertainties in biomass burning/fire emissions from emissions inventories [2,4,5,8,11,12]. GFEDv4.1s, which does not make cloud-adjustments for obscured fires, may underestimate emissions under severe haze events. FINNv1.5 emissions inventory, which assumes a high percentage (>75%) of burned area per pixel, may overestimate small fires [7]; GFASv1.2 and FEERv1.0-G1.2, which use a coarse-resolution (0.5°-1°) land use and land cover (LULC) map, may bias emissions in areas with complex LULC, such as India [4]. QFEDv2.5r1, which enhances aerosol emissions with a constant global scaling factor for each LULC, may not accurately represent regional smoke emissions [13]. SAGE-IGP, while giving a more realistic state- and seasonal-level budget for dry matter emissions than other global emissions inventories, has its own limitations and uncertainties in constructing emissions (e.g. use of disproportionate survey data for validation) [11]. Each emissions inventory has its own strengths and limitations, no studies on which emissions inventory most accurately represents Indian crop residue burning have yet been completed.

One contribution of this work is the quantification of the relative contribution to air quality impacts due to emissions in different locations and times. While large discrepancies in total emissions estimate exist in emissions inventories, the fractional contribution of agricultural fire emissions sources to total air quality impacts in India are consistent (Supplementary Fig. 23). Supplementary Fig. 23 shows the spatial (grid) attribution of $PM_{2.5}$ exposure to post-monsoon agricultural emissions among six emissions inventories. Fire emissions in Punjab (southeast part) and Haryana are consistently the largest contributor to the enhancement of $PM_{2.5}$ exposure throughout the inventories. Specifically, these two states together contribute to more than 75% of annual $PM_{2.5}$ exposure enhancement due to post-monsoon agricultural residue burning. Specifically, Punjab alone is responsible for more than 60% of total air quality impacts from crop residue burning, regardless of meteorology variability and choice of emissions inventory (Supplementary Fig. 24). Fractional contribution of $PM_{2.5}$ exposure due to emissions in Haryana has larger interannual variations (potentially due to meteorological variability) in bottom-up inventories (GFEDv4.1s, FINNv1.5, SAGE-IGP) than top-down inventories (GFASv1.2, QFEDv2.5r1, FEERv1.0-G1.2). Despite potential variations in estimates (in absolute numbers) of air quality impacts (e.g. $PM_{2.5}$ exposure, premature deaths), relative contribution from emissions in space and time remain consistent ($\pm$5%) in global emissions inventories.

Previous studies have suggested that integration of both bottom-up (e.g. burned area) and top-down (e.g. active fire) approaches may reduce the uncertainties in emissions inventories [4]. The latest GFEDv4s emissions inventory has adopted a hybrid approach of using active fire counts for small fire boost, which may less underestimate fire emissions. Using GFEDv4s and the most recent IERs and baseline mortality rates as well as additional adjoint simulations, we update annual premature deaths due to $PM_{2.5}$ exposure from agricultural residue burning in India as 44,000-98,000 from 2003 to 2019, specifically 60,000 (50,000-70,000) for 2015, consistent with GBD India Special Report 2018 using the same emissions inventory, which estimated 66,000 (57,000-79,000) premature deaths attributable to ambient

$PM_{2.5}$ from open agricultural burning in India in 2015 [14].

The surface $PM_{2.5}$ derived from satellites include biogenic, geogenic, primary anthropogenic, and secondary aerosols that form as a result of chemical precursors such as NO_x and SO_x [15]. Emissions of anthropogenic, primary $PM_{2.5}$ alone have previously been estimated to have an uncertainty of up to 30% for South Asia [16]. Since we consider only the impacts of agricultural primary $PM_{2.5}$ emissions, errors that arise from inaccuracies in other anthropogenic emissions will not affect the accuracy of our estimates.

1.2 Satellite errors, model limitations and comparison to ground-based $PM_{2.5}$

There are several potential reasons for the discrepancies between satellite AOD/$PM_{2.5}$ and GEOS-Chem modeled AOD/$PM_{2.5}$. These include satellite retrieval errors, such as surface reflectance estimation and cloud screening, and model limitations due to such factors as inaccurate aerosol optical properties, incomplete emissions inventories, and inaccurate meteorological fields [4,5]. Previous studies using GEOS-Chem to simulate South Asia/India concluded that the greatest model inaccuracies are due to incomplete or outdated emissions inventories [4,5,11]. The discrepancy is also likely driven by model uncertainties in simulating aerosols from other sources. Apart from agricultural burning smoke, other significant components of India's aerosol burden include local dust lofted under dry and hot conditions; long-range transport of dust from deserts in Asia and Africa; and anthropogenic pollutants such as NO_x and SO_2 [15]. David et al. (2019) [17] found that GEOS-Chem underestimates dust aerosols in western and northwestern India and overestimates anthropogenic aerosols in south and northeast India, which may explain the discrepancies in our comparison. Despite this, among total aerosols our focus is those related to fine-mode aerosols from agricultural burning. Existing research shows the simulated fine-mode AOD is captured by GEOS-Chem when compared against measurement data [17]. Furthermore, the computation of adjoint sensitivities for black and organic carbon is not affected by emission inventories used in forward modeling.

The Central Pollution Control Board (CPCB) of India provides hourly records of surface $PM_{2.5}$ at 197 monitoring stations throughout India, including 37 stations in Delhi. We use a separate and updated forward GEOS-Chem model (version 13.0.2) and run additional 23 pairs of simulations for each year between 2003 and 2019. We collect available ground-based PM2.5 from CPCB (https://app.cpcbccr.com/ccr/#/caaqm-dashboard-all/caaqm-landing/caaqm-data-availability) and US Embassy Air Quality Monitors (https://www.airnow.gov/international/us-embassies-and-consulates/#India$New_Delhi) for 2018 and 2019, to which we compare modeled $PM_{2.5}$. To better quantify the sources of disagreement between model and observations and their likely effect on our conclusions, we first performed an additional set of quality control procedures and additional comparisons of modeled and observed $PM_{2.5}$. We have also evaluated model performance by statistical metrics at the individual city level (Supplementary Fig. 15).

The data quality of observed $PM_{2.5}$ from Central Pollution Control Board (CPCB) varies significantly at different locations and times (Supplementary Fig. 15, Supplementary Data 6). For example, 89% of the daily monitored data for Alipur (Delhi) were not available during the 2018 fire season; although zero concentrations are not likely to occur during fire seasons in cities in northeastern India, Amritsar (Punjab) recorded consecutive zero concentrations for 20 days in October in 2018. Thus, similar to Kumar et al., 2020 [18], we have taken the following steps to assure the quality of observed data.

First, we filter observations that are below 10 $\mu g\ m^{-3}$ and above 1,500 $\mu g\ m^{-3}$, as values being too low or too high that likely resulted from instrument malfunction [18]. Second, we keep those stations where observations are available

over 75% of the time during fire season (i.e. 45 days). With these steps, out of 68 in 2018 and 110 cities in 2019 that were originally used in model validation, 50% and 31% respectively are filtered out. Here we show the model performance in terms of Pearson correlation coefficient (R) and mean bias (MB) for the remaining cities (Supplementary Fig. 15, Supplementary Data 6).

Shown in Supplementary Fig. 15, the GEOS-Chem model captures the temporal variability and the magnitude on local scale. The modeled $PM_{2.5}$ tends to be at the lower bound (OBS min) compared with CPCB observations (OBS avg) in 2018 and 2019. We clarify that we only simulate black carbon (BC) and organic carbon (OC) as $PM_{2.5}$, which is the primary component of fire smoke and is naturally different from the measured $PM_{2.5}$, which consists of other components and secondary $PM_{2.5}$ (e.g. sulfate aerosol) from fire and non-fire emissions sources. However, the modeled daily $PM_{2.5}$is within the 24-hour maximum and minimum observations at more than 90% of locations.

As shown in Supplementary Fig. 15, the modeled carbonaceous $PM_{2.5}$ generally correlates well with observed total $PM_{2.5}$ (R~0.4-0.8), including Delhi, Patiala and Chandigarh (Punjab), Kaithal and Faridabad (Haryana). This correlation is of course not perfect. For example, the R values in 15% (3%) cities in 2018 (2019) are below 0.2. In addition to model limitations which we have discussed in SI Section 3, random fluctuations in the observations that still exist after filtering may also affect the validation statistics [18]. For instance, a few stations in Haryana showed high $PM_{2.5}$ values (>900 μg m^{-3}) for 5–6 hours right after the missing values (−999.0) were reported, which is not filtered and may have affected the validation [20].

Despite the discrepancies compared with CPCB measurements at a few cities (e.g. R~0.3 in Panchkula, Punjab), we focus on the average increased air quality impacts across India due to burning. Therefore, local biases are unlikely to significantly impact our estimates for population exposure and subsequent health impacts. Overall, the GEOS-Chem model performs reasonably well in capturing the broad spatial pattern and magnitude of $PM_{2.5}$ and AOD in India comparing with observations from various sources (Figure 6, Supplementary Figures 11-14). These results suggest that the GEOS-Chem model can provide useful results for air quality studies.

2. IER and other concentration response functions

The premature mortality estimates in this study depend on the specific choice of concentration response function (CRF). Various CRFs have been developed to quantify excess deaths attributable to $PM_{2.5}$ exposure [19]. For example, the log-linear model (LL), the first relative risk function, was used by the US EPA (2012) [20] to estimate benefits of reducing air pollution in the US; the Global Exposure Mortality Model (GEMM) was developed to represent potentially non-linear associations between exposure and mortality and has been used by the Global Burden of Disease (GBD 2017, Burnett et al., 2018) [21,22]; a Meta Regression-Bayesian, Regularized, Trimmed (MR-BRT) spline was recently developed by GBD 2019 [23] to incorporate the latest observational cohort and case-control studies on disease burden. As with GBD practice, we use the latest available integrated exposure-response (IER) function to estimate the relative risk (RR) of premature mortality due to exposure to outdoor $PM_{2.5}$ concentrations. Each of these functions has strengths and limitations that may have different implications depending on specific objectives [19].

The IER function estimates the population averaged premature mortality due to an increase in long-term (annual mean) air pollution rather than immediate deaths due to short-term spikes. This approach has been used in a number of studies to evaluate long-term health impacts of biomass burning episodes [13,24-27]. Such a method is designed for annual average calculations, and do not give a time series of impact changes over time. During severe haze events of rice

residue burning season, the 24-hour mean $PM_{2.5}$ concentrations are 20-35 times above the safety guideline set by WHO (25 µg m^{-3}) and can occasionally reach 1,000 µg m^{-3} [28]. It is possible that such a spike of $PM_{2.5}$ exposure from crop residue burning has short-term health impacts that leads to premature mortality, for example, acute lower respiratory infection.

2.1 Log-linear (LL) model

Here we quantify the daily all-cause mortality to changes in population exposure to outdoor $PM_{2.5}$ by adopting a log-linear function from Atkinson et al (2014) [29]:

$$\mathrm{RR} = e^{\beta\chi} \tag{14}$$

where χ is the daily $PM_{2.5}$ concentration, and β is 1.04% (95% CI: 0.52%-1.56%), an increase in all-cause mortality for every 10 µg m^{-3} increase in same-day $PM_{2.5}$ concentrations. We also estimate premature mortality cause by cardiovascular and respiratory diseases assuming a β of 0.84% (95% CI: 0.41%-1.28%) and a β of 1.51% (95% CI: 1.01%-2.01%), respectively.

We take the annual baseline incidence rate for all-cause mortality $I(\chi_{\mathrm{base}})$ and cause-specific mortality at $PM_{2.5}$ level χ_{base} (observed baseline daily mean) from GBD 2019 [23] and divide by 365 to obtain daily baseline incidence of mortality.

The daily change in premature mortality ΔM_h in a grid cell (i, j) for a specific cause h from crop residue burning is estimated using:

$$\Delta M_h = \rho \cdot I_h(\chi_{\mathrm{base}}) \cdot \frac{\mathrm{RR}_h(\chi_{\mathrm{base}} + \Delta\chi) - \mathrm{RR}_h(\chi_{\mathrm{base}})}{\mathrm{RR}_h(\chi_{\mathrm{base}})} \tag{15}$$

where $\Delta\chi$ is an increase in daily $PM_{2.5}$ concentrations, ρ is the affected population.

A Taylor expansion of the numerator in Supplementary Equation (15) gives:

$$\mathrm{RR}_h(\chi_{\mathrm{base}} + \Delta\chi) - \mathrm{RR}_h(\chi_{\mathrm{base}}) = \Delta\chi \left(\frac{\partial \mathrm{RR}_h}{\partial \chi}\right)_{\chi=\chi_{\mathrm{base}}} + O(\Delta^2\chi) \tag{16}$$

Combining Supplementary Equations (14) and (16), and neglecting the 2nd and higher order terms of Supplementary Equation (16),

$$\frac{\mathrm{RR}_h(\chi_{\mathrm{base}} + \Delta\chi) - \mathrm{RR}_h(\chi_{\mathrm{base}})}{\mathrm{RR}_h(\chi_{\mathrm{base}})} = \beta\Delta\chi \qquad \text{S17}$$

Combining Supplementary Equations (14)-(17), we obtain:

$$\Delta M_h = \rho \cdot I_h(\chi_{\text{base}}) \cdot \beta \Delta \chi \tag{18}$$

Given that $\rho \Delta \chi = (S \circ E)$ in the adjoint model, we finally obtain:

$$\Delta M_h = \beta \cdot I_h(\chi_{\text{base}}) \cdot (S \circ E) \tag{19}$$

where S is the adjoint sensitivity, and E is the agricultural $PM_{2.5}$ emissions.

Thus, the total daily premature deaths are:

$$\Delta M = \sum_{ijt} \Delta M_h \tag{20}$$

Using Supplementary Equation (20), we estimate the contribution of short-term $PM_{2.5}$ exposure from crop residue burning to cause-specific daily premature deaths as shown in Supplementary Data 9. We estimate the all-cause premature deaths based on the short-term approach as 30,000 (95% CI: 24,000-35,000), which is approximately 56% less than the estimate using the IER function. However, the mathematical forms to estimate premature mortality of the two approaches are similar (Supplementary Equations (13) and (19)), indicating that using a different function would only change the absolute number of estimated premature deaths but not the fractional contribution by location and time.

2.2 Global Exposure Mortality Model (GEMM)

We also use the Global Exposure Mortality Model (GEMM) to calculate the mortality burden due to long-term (annual) exposure to $PM_{2.5}$ [21]. The GEMM has the form:

$$\log(\text{RR}) = \theta \log\left(\frac{\chi}{\alpha + 1}\right) / (1 + e^{\frac{-(\chi - \mu)}{\tau r}}) \tag{21}$$

where θ, μ, τ are unknown parameters, r is the range in the pollutant concentrations, χ is the observed $PM_{2.5}$ concentration. We use parameters adopted from GBD 2019 [23]. The GEMM curves are shown in Supplementary Fig. 25 and repeat calculations in a similar manner with the IER and LL models. We estimate 99,000 (95%CI: 84,000-110,000) premature deaths annually due to agriculture fires. This number is 45% higher than the average value using the IER method. Such a significant increase is also found in global attributable mortality based on the GEMM model, which was found to be twice as much as the IER-based estimates (Burnett et al., 2018, Burnett and Cohen 2020), partly because the GEMM models all-natural cause mortality and the non-communicable disease + lower respiratory infection (NCD + LRI) mortality rates used in the GEMM are much higher than the sum of cause-specific mortality rates as used in the IER (Burnett and Cohen 2020).

2.3 Meta Regression-Bayesian, Regularized, Trimmed (MR-BRT) splines

The GBD 2019 introduced a Meta Regression-Bayesian, Regularized, Trimmed (MR-BRT) spline for the estimation of attributable mortality [30]. MR-BRT curves use splines with Bayesian priors which avoid over-estimation of risks

at high exposure levels. We adopt the MR-BRT curves from GBD 2019 (as shown in Supplementary Fig. 26) and because it is difficult to apply the adjoint sensitivities to Bayesian priors, we fit a spline function using the curves, from which we obtain four parameters and their range. Using a Monte Carlo approach, we estimate 81,000 (95%CI: 67,000-95,000) premature deaths attributable to crop residue burning, which is 17% higher than the IER-based estimates.

Using a different CRF may significantly change the estimate of premature deaths, reflecting uncertainty in the true size of mortality burden. Burnett and Cohen., 2020 [19] reviewed different CRFs and recommend IER as a useful and preferred method. The LL model assumes a linear relationship between exposure and mortality risk, which may not hold true for all outcomes. Thus, the LL model is primarily used in studies on populations exposed to lower concentrations. The estimate of relative risk using the GEMM model was found highly sensitive to high pollution concentrations, which is the case for India, therefore it should be carefully applied to countries like India. The MR-BRT model, while incorporates the latest cohort epidemiological and experimental studies, the practicality and reliability of this new model have not been formally tested.

The IER model has evolved and been improved since its introduction in 2010. The IER has been extensively applied by the Global Burden of Disease (GBD), the World Health Organization (WHO), the World Bank, the United States Environmental Protection Agency (US EPA) at global, national and regional scales [19]. Although not specifically developed for India, the applicability of the IER in the Indian context has been reviewed and supported by the Steering Committee on Air Pollution and Health Related Issues of the Indian Ministry of Health and Family Welfare (GBD Working Group 2018) [14]. We believe the IER is a useful method to quantify exposure-attributable mortality risks.

2.4 Location-specific baseline mortality rate

We also recognize that India as a country with varied socio-economic conditions, a uniform country-level baseline mortality rate (BMR) may not be the best practice for calculating the attributable deaths. Chowdury and Dey, 2016 [31] suggested using a state-specific BMR adjusted by a function of GDP in India. GDP-based proxy of BMR follow the formula

$$G = \begin{cases} a(1+y_h)^b & h = \text{IHD} \\ a{y_h}^b & h = \text{COPD, CeVD} \end{cases} \tag{22}$$

where G is state-specific GDP per capita in India, y is the baseline mortality rate, and a, b are disease-specific constants provided by Chowdhury et al., 2016 [31]. Using S22, we find a 10%-12% increase in calculated early deaths (Supplementary Fig. 27), which is consistent with Chowdhury et al., 2016 (15% increase) [31]. While it is ideal to use a BMR that accounts for the socio-economic heterogeneity in a country like India, a mathematical formula between GDP and state-specific BMR is only found for three diseases (i.e. COPD, IHD and CeVD), and such a GDP proxy of BMR does not take age, gender and other demographic factors into account. Robust statistics and empirical evidence are needed to test the non-linear relationship (Supplementary Equation (22)) for approximating BMR. Therefore, it is not clear whether GDP per capita can fully represent the spatial baseline mortality variation across India due to socio-economic heterogeneity. As additional studies on BMR grows, we will continually re-examine the practicality of a uniform BMR and make possible updates to the application of CRF with respect to socioeconomic variations.

Supplementary figures

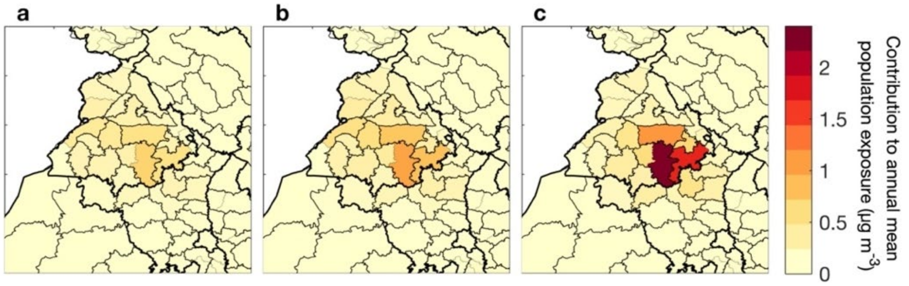

Figure 1. Contribution to increased $PM_{2.5}$ exposure over different dispserse populations from agricultural residue burning emissions. a. District-wise contribution averaged over “normal” years to annual mean population weighted exposure to outdoor $PM_{2.5}$ from agricultural burning for population over all India. b. Same as a. but for population over urban areas (population density > 400 people per km^2). c. Same as a. but for population over densely populated areas (population density > 1,000 people per km^2) in northwest India (73°E-78°E, 28°N-33°N). Population map corresponding to each sub-plot is provided in Supplementary Fig. 31. India administrative maps are obtained from the Database of Global Administrative Areas, Version 4.1 (https://gadm.org/data.html). Coast outlines are plotted based on data in MATLAB [32].

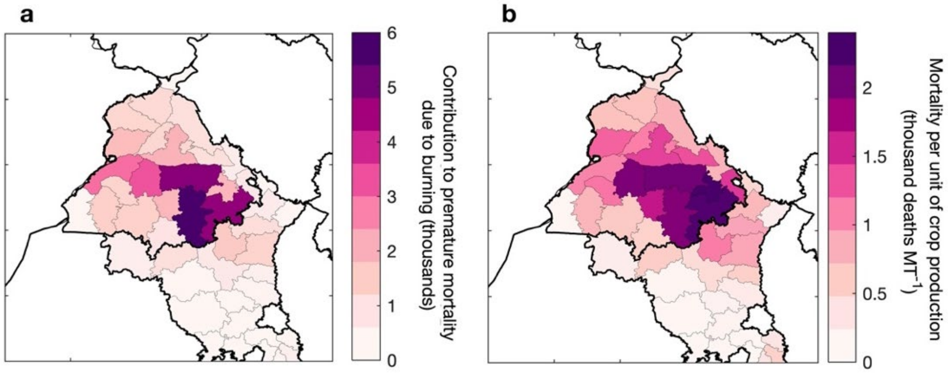

Figure 2. Contribution of each district’s (in Punjab and Haryana) residue burning emissions to premature deaths across India. a. The absolute amount of mortalities across all of India that resulted from agricultural burning in Punjab and Haryana districts. b. Mortalities in a. divided by the total annual crop production from the same region. The region shown is northwest India (73°E-78°E, 28°N-33°N). . India administrative maps are obtained from the Database of Global Administrative Areas, Version 4.1 (https://gadm.org/data.html). Coast outlines are plotted based on data in MATLAB [32].

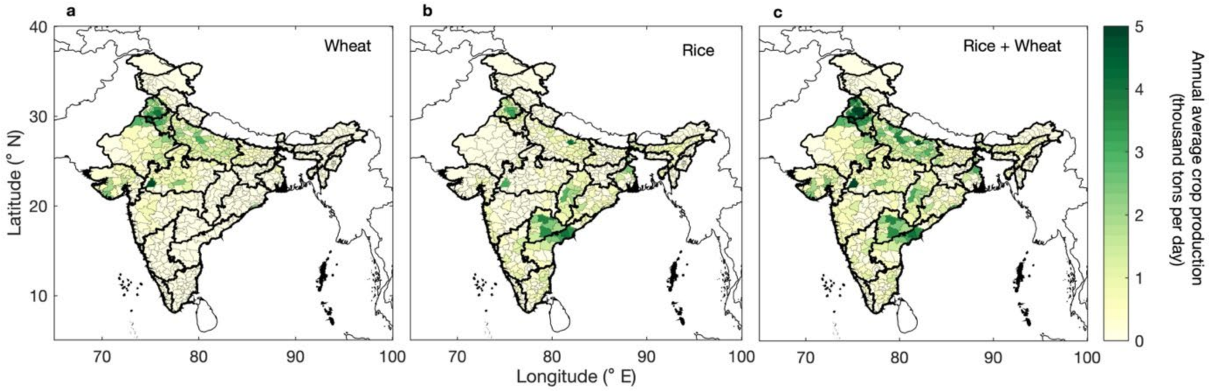

Figure 3. Annual rice and wheat production by district in India. a. Annual average (1997-2014) district-wise wheat production. b Same as a. but for rice production. c. Annual rice and wheat production (a + b) in India. Rice residue burning is not widely practiced in some large rice producers in south India such as Andhra Pradesh and Telangala because these states, unlike Punjab and Haryana, do not have a rice-wheat rotation and thus have abundant time to deal with crop residue between harvesting and planting season. Data source: India Open Government Data Platform (http://data.gov.in/). India administrative maps are obtained from the Database of Global Administrative Areas, Version 4.1 (https://gadm.org/data.html). Coast outlines are plotted based on data in MATLAB [32].

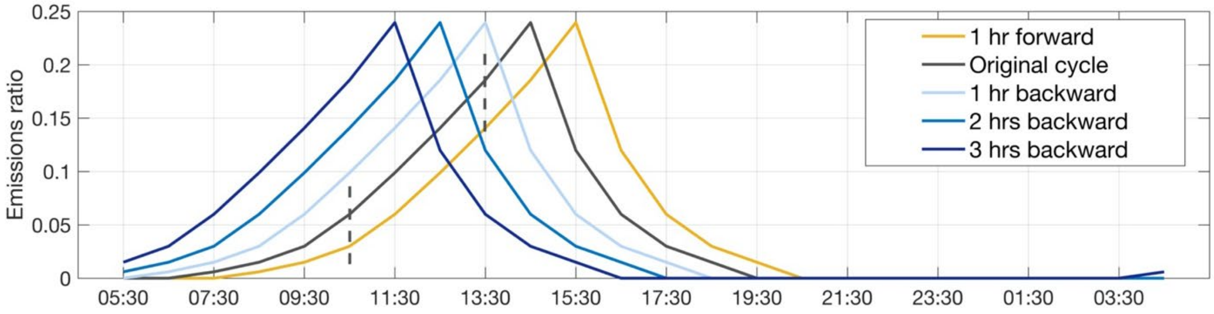

Figure 4. Diurnal cycles of local agricultural burning emissions in residue burning seasons, where emissions at 13:30 LT are three times as large as those at 10:30 LT, with a peak at 14:30 LT, based on burning cycles in India and India MODIS fire detection analysis during crop residue burning seasons by Vadrevu et al., 2011 [33].

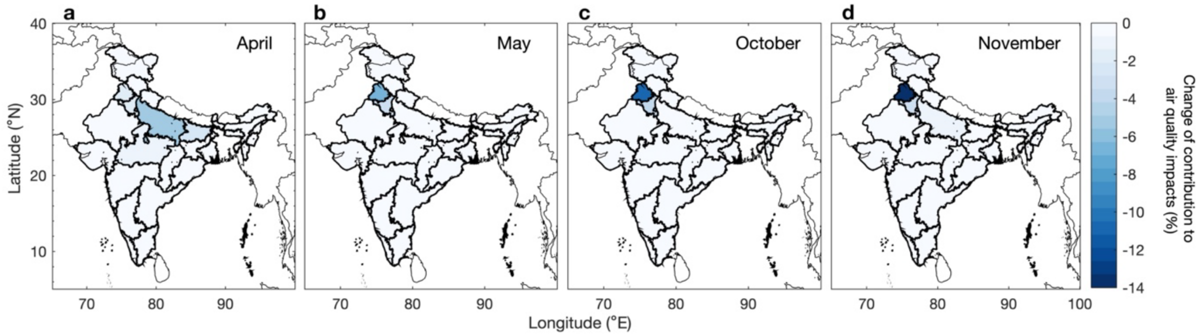

Figure 5. Percentage changes of contribution to air quality impacts by burning 2 hours earlier. a. Percentage change of contribution to air quality impacts due to state-wise burning 2 hours earlier in April. b. Same as a but for May. C. Same as a but for October. d. Same as a but for November. India administrative maps are obtained from the Database

of Global Administrative Areas, Version 4.1 (https://gadm.org/data.html). Coast outlines are plotted based on data in MATLAB [32].

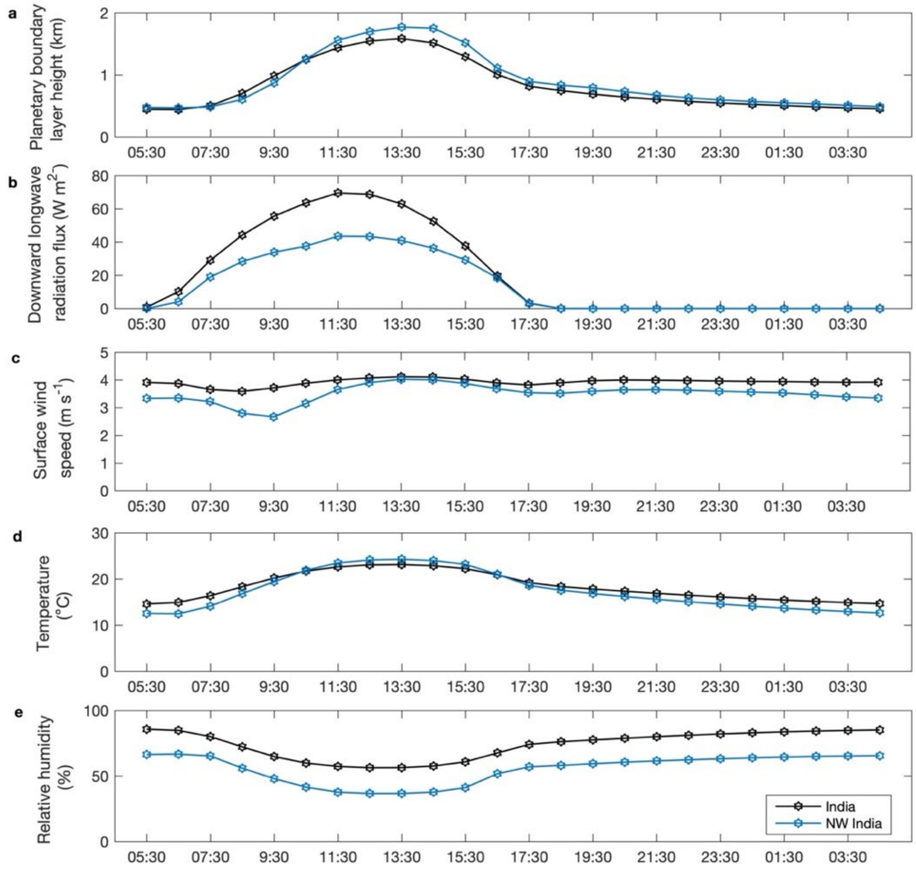

Figure 6. Local time (LT) diurnal cycles of meteorological parameters in India. a. hourly planetary boundary layer height (km) averaged over Oct 1st-Nov 30th in India (black) and northwestern India (blue). b. Same as a but for hourly surface downwelling diffuse flux (W m^{-2}). c. Same as a but for hourly surface wind speed (m s^{-1}). d. Same as a but for hourly 2m air temperature (^{o}C). e. Same as a but for hourly relative humidity (%). Data is for the year 2021 and is taken from the MERRA-2 reanalysis (https://disc.gsfc.nasa.gov/).

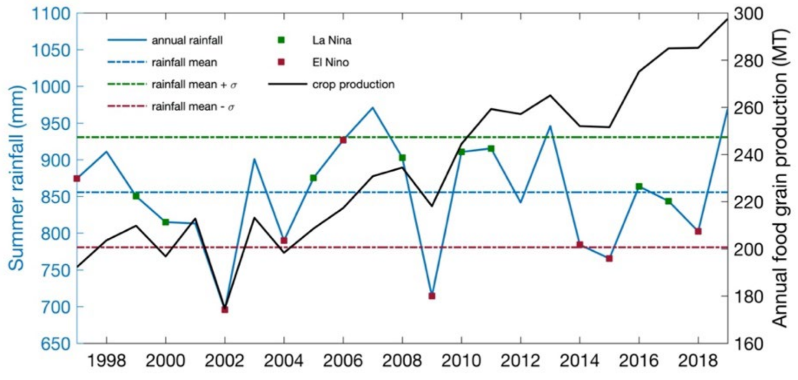

Figure 7. Annual monsoon rainfall over India from 1997 to 2019 and total food grain production (rainfall data acquired from Open Government Data Platform in India, https://data.gov.in, crop data obtained from Indiastat,

https://www.indiastat.com/data/agriculture. Years with rainfall below the red dashed line are classified as drought years, those with rainfall above the green line are classified as flood years, the rest are normal rainfall years.

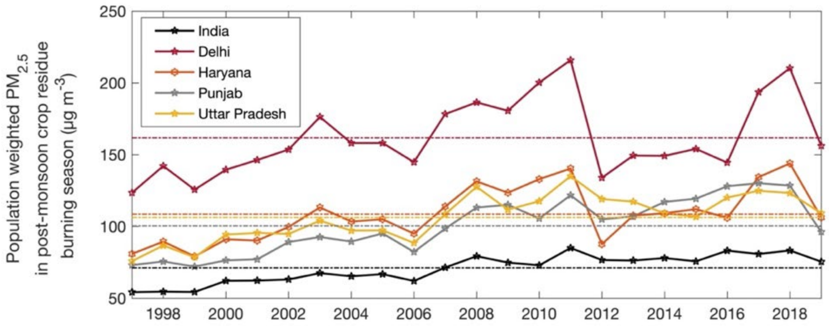

Figure 8. GEOS-Chem modeled daily mean population-weighted $PM_{2.5}$ in India, Delhi, Haryana, Punjab, and Uttar Pradesh during Oct-Nov from 1997 to 2019.

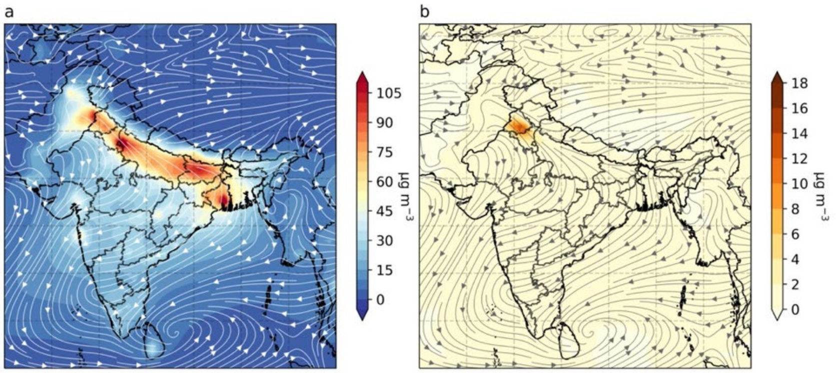

Figure 9. a. GEOS-Chem modeled daily-mean surface $PM_{2.5}$ in μg m^{-3} during post-monsoon residue burning season from 1997 to 2019. b. . GEOS-Chem modeled enhanced $PM_{2.5}$ (full emissions – agricultural off) in μg m^{-3} during post-monsoon residue burning season from 1997 to 2019. Streamlines denote daily mean 500m wind field of same period taken from MERRA-2 reanalysis (https://disc.gsfc.nasa.gov/). India administrative maps are obtained from the Database of Global Administrative Areas, Version 4.1 [50]. Coast outlines are plotted based on data in Python Matplotlib Basemap Toolkit, Version 1.2.1.

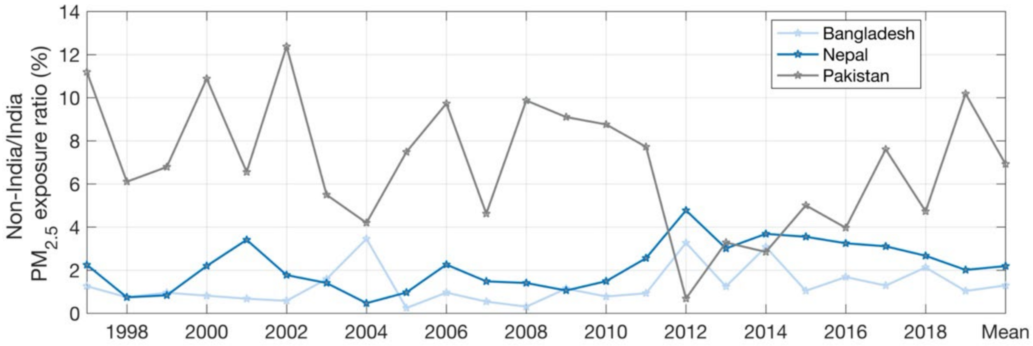

Figure 10. Ratio of $PM_{2.5}$ exposure enhancement in neighboring countries (i.e. Bangladesh, Nepal, Pakistan) to that in India due to post-monsoon crop residue burning emissions in India.

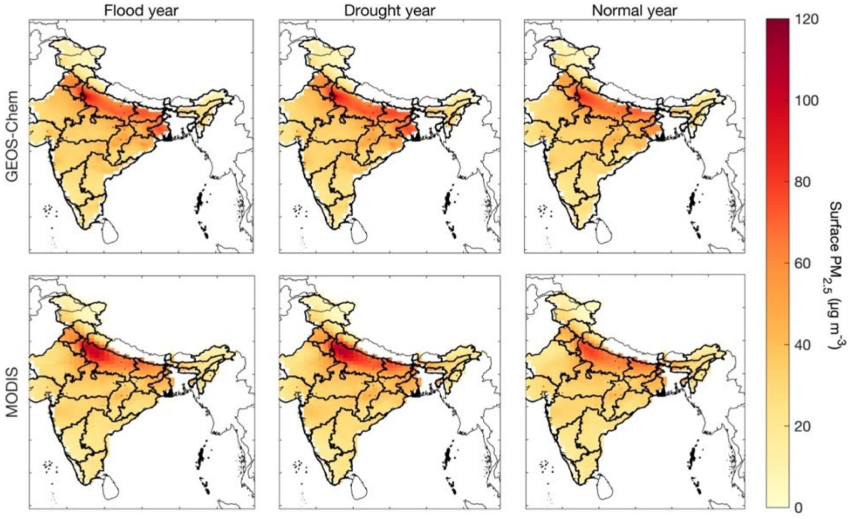

Figure 11. Comparison of surface $PM_{2.5}$ between GEOS-Chem forward simulations and MODIS satellite-derived estimates for (a) drought, (b) flood and (c) normal years. Satellite-derived $PM_{2.5}$ is available at https://sedac.ciesin.columbia.edu/data/set/sdei-global-annual-gwr-pm2-5-modis-misr-seawifs-aod-v4-gl-03. India administrative maps are obtained from the Database of Global Administrative Areas, Version 4.1 (https://gadm.org/data.html). Coast outlines are plotted based on data in MATLAB [32].

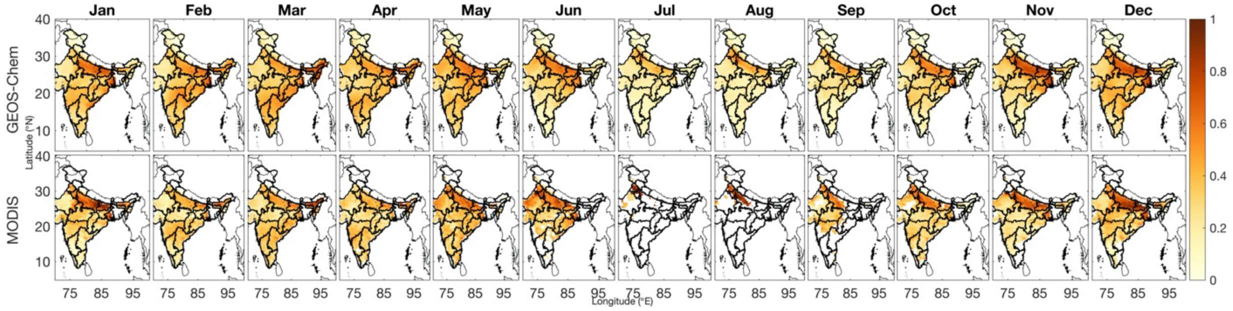

Figure 12. AOD comparison between GEOS-Chem and MODIS (MODIS data from June to September is partially missing). MODIS AOD data is available at https://ladsweb.modaps.eosdis.nasa.gov/archive/allData/61/. India administrative maps are obtained from the Database of Global Administrative Areas, Version 4.1 (https://gadm.org/data.html). Coast outlines are plotted based on data in MATLAB [32].

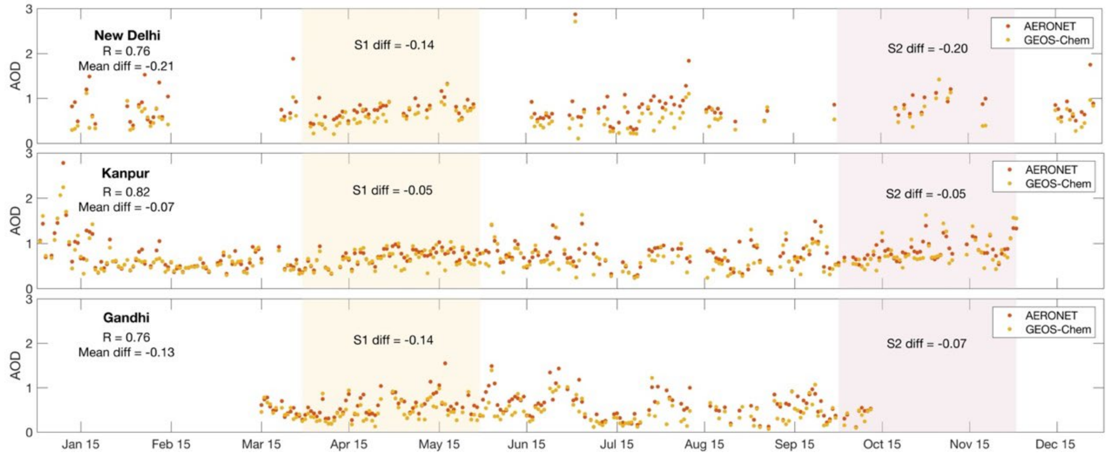

Figure 13. AOD comparison between GEOS-Chem and three AERONET sites (New Delhi, Kanpur, and Gandhi College) in India (only available AERONET data points are compared). The data for AERONET AOD in India is available at https://aeronet.gsfc.nasa.gov/cgi-bin/draw_map_display_aod_v3.

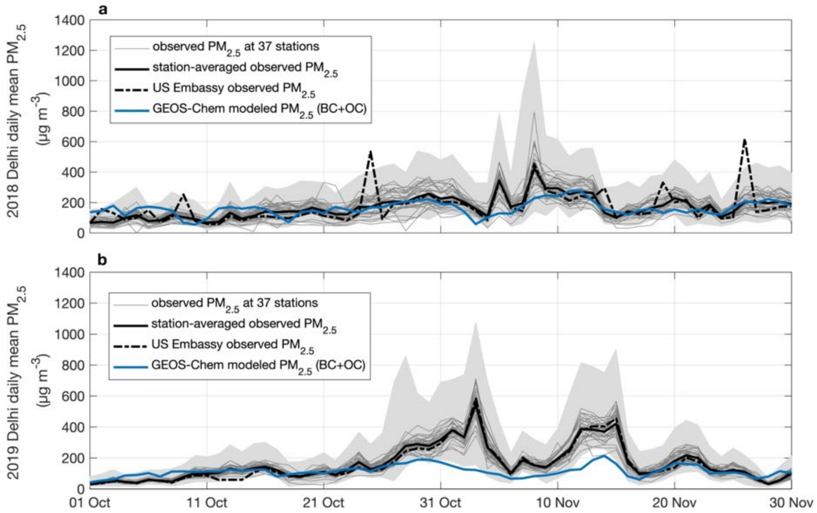

Figure 14. Comparison between GEOS-Chem modeled and observed $PM_{2.5}$ in Delhi. a. GEOS-Chem modeled (blue line) and Central Pollution Control Board (CPCB, solid grey and bold black lines)/US Embassy (dashed black lines) in 2018. b. Same as a but for 2019. Shaded area denotes the daily (24-hour) maximum/minimum CPCB observed $PM_{2.5}$. CPCB $PM_{2.5}$ data is available at https://app.cpcbccr.com/ccr/#/caaqm-dashboard-all/caaqm-landing/caaqm-data-availability. US Embassy $PM_{2.5}$ data is available at https://www.airnow.gov/international/us-embassies-and-consulates/#India$New_Delhi.

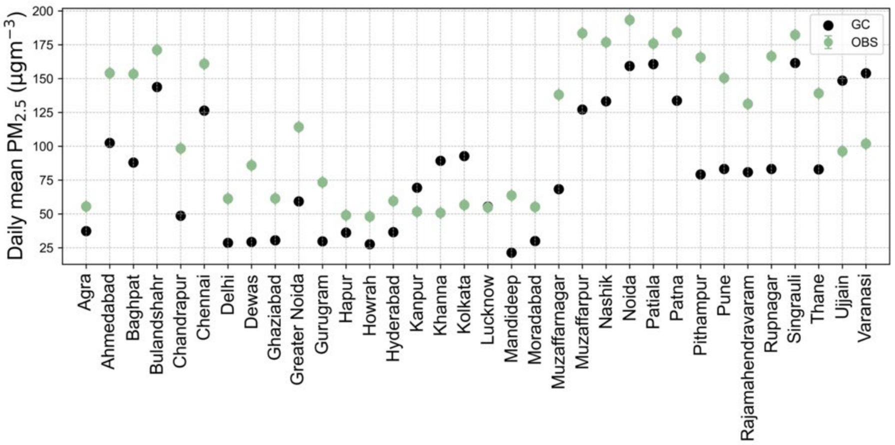

Figure 15 a. Comparison of daily mean $PM_{2.5}$ during post-monsoon residue burning season between GEOS-Chem (BC+OC) outputs and 24-hour mean, max, min CPCB/US Embassy observations at 34 qualified cities in 2018. Name of cities and $PM_{2.5}$ values for each day are provided in Supplementary Data 6.

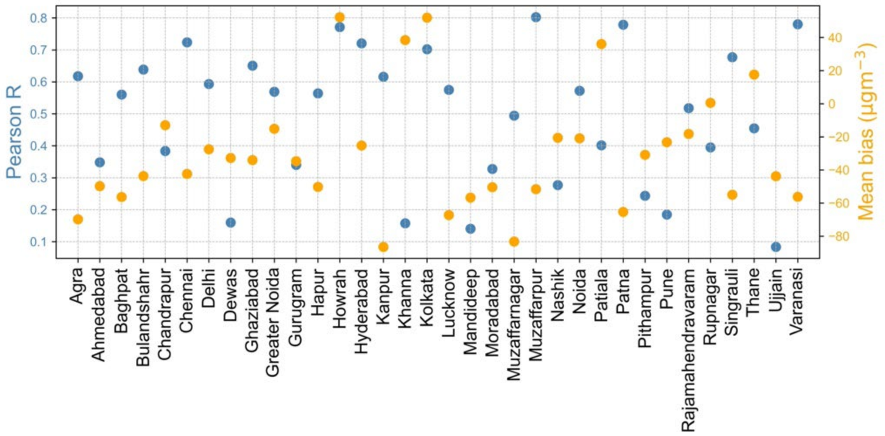

Figure 15 b. Pearson R and mean bias between daily mean $PM_{2.5}$ during post-monsoon residue burning season between GEOS-Chem (BC+OC) outputs and 24-hour mean CPCB/US Embassy observations at 34 qualified cities in 2018. Name of cities and $PM_{2.5}$ values for each day are provided in Supplementary Data 6.

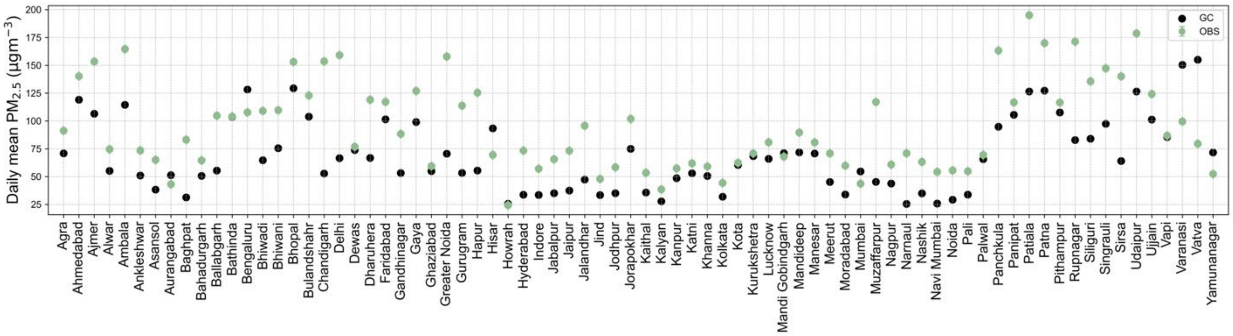

Figure 15 c. Comparison of daily mean $PM_{2.5}$ during post-monsoon residue burning season between GEOS-Chem (BC+OC) outputs and 24-hour mean, max, min CPCB/US Embassy observations at 76 qualified cities in 2019. Name of cities and $PM_{2.5}$ values for each day are provided in Supplementary Data 6.

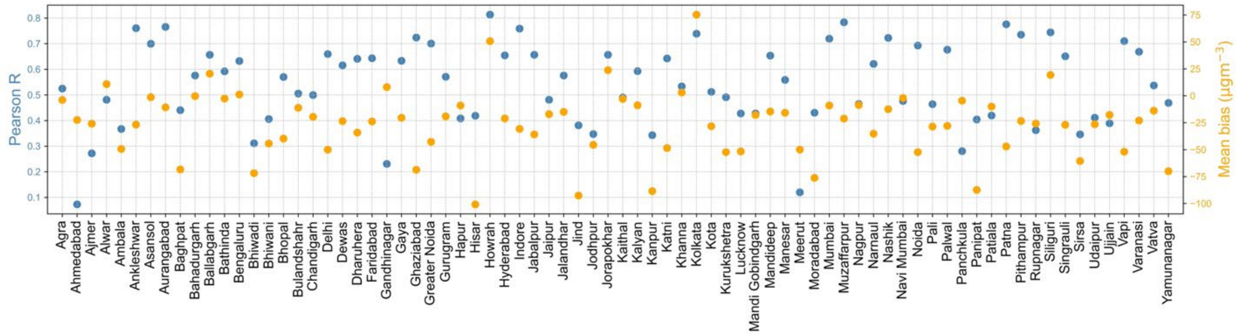

Figure 15 d. Pearson R and mean bias between daily mean $PM_{2.5}$ during post-monsoon residue burning season between GEOS-Chem (BC+OC) outputs and 24-hour mean CPCB/US Embassy observations at 67 qualified cities in 2019. Name of cities and $PM_{2.5}$ values for each day are provided in Supplementary Data 6.

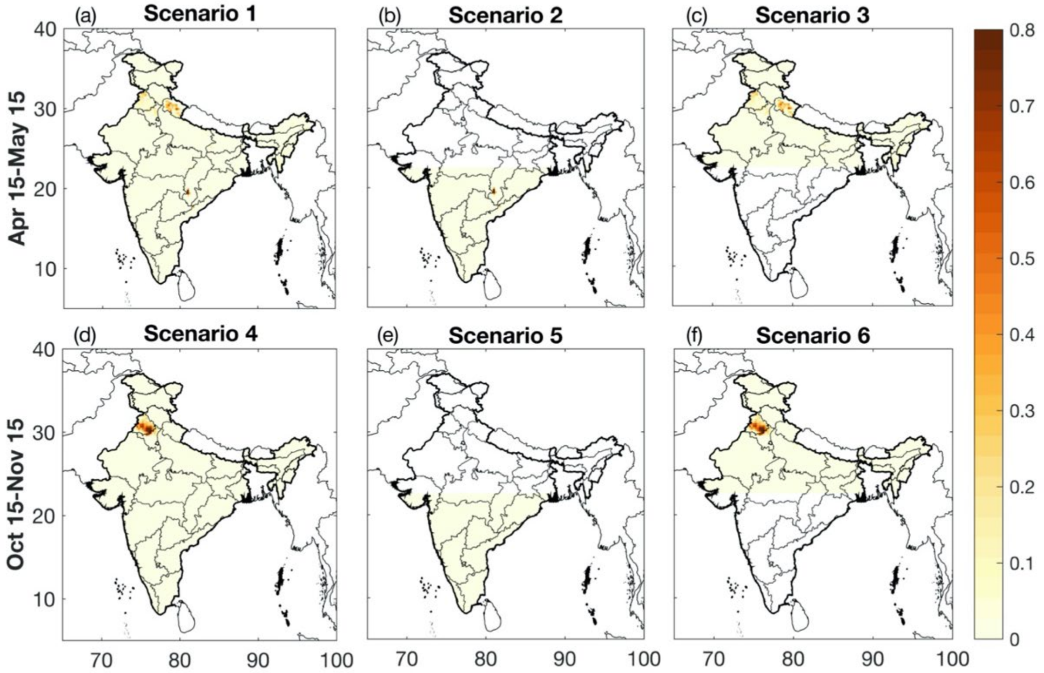

Figure 16. Corresponding to Supplementary Data 7, biomass burning emission scenarios based on GFED4.1s. Scenario 1: Biomass burning emissions (in thousand tons per day) turned on for all India from April 15-May 15, 2009; Scenario 2: Biomass burning emission turned on for southern India from April 15-May 15, 2009; Scenario 3: Biomass burning emission turned on for northern India from April 15-May 15, 2009; Scenarios 4-6, same as Scenarios 1-3 but from October 15-November 15, 2009. India administrative maps are obtained from the Database of Global Administrative Areas, Version 4.1 (https://gadm.org/data.html). Coast outlines are plotted based on data in MATLAB [32].

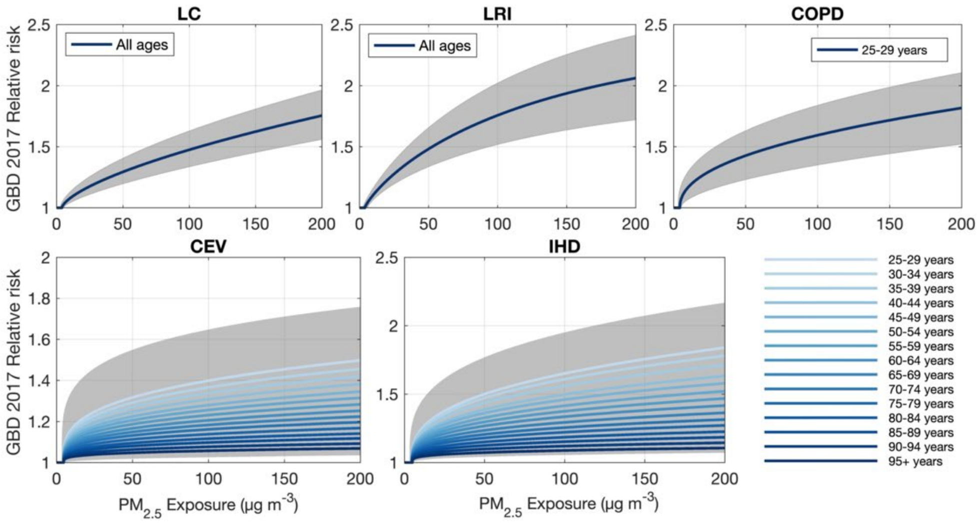

Figure 17. GBD 2017 IER curves (India specific) with age adjustments and older age groups. (Data obtained from http://ghdx.healthdata.org/record/ihme-data/gbd-2017-burden-risk-1990-2017)

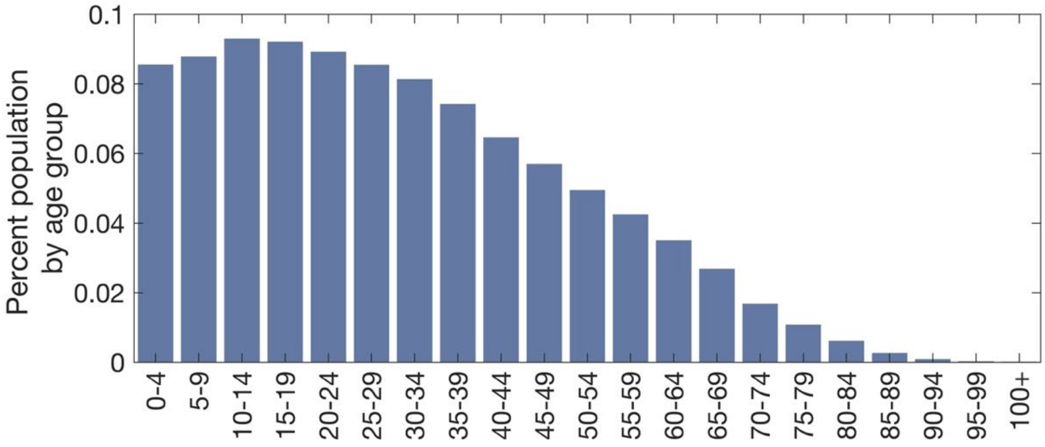

Figure 18. Percent population by age groups at five-year interval in India from 2005 to 2019. Data source: United Nations, Department of Economic and Social Affairs, Population Division. World Population Prospects: The 2019 Revision. (Data obtained from https://population.un.org/wpp/Download/Standard/Population/)

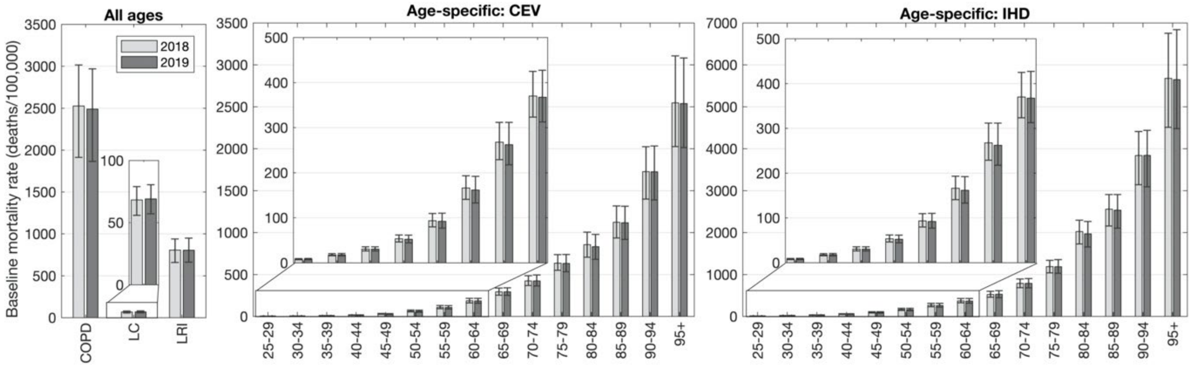

Figure 19. GBD 2019 India- and disease-specific baseline mortality rates (data obtained from http://ghdx.healthdata.org/gbd-results-tool)

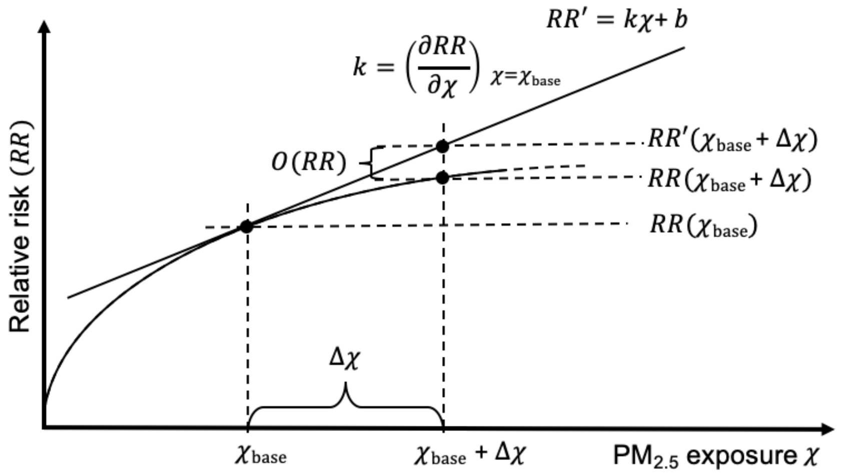

Figure 20. Linearization and error of RR estimation

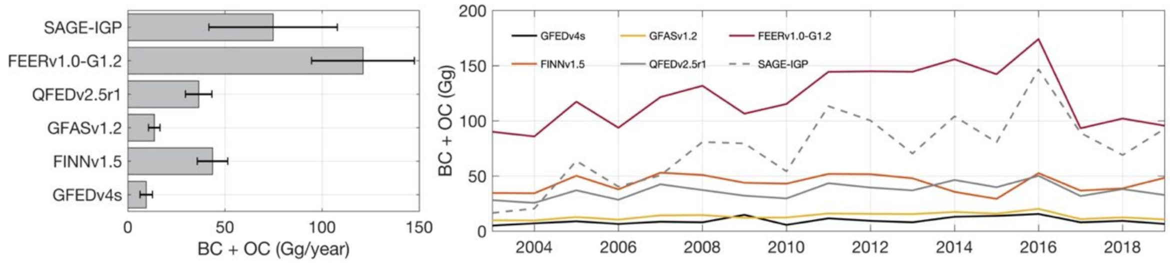

Figure 21. BC and OC emissions comparison accross emissions inventories. (left) Annual-mean ($\pm\sigma$) and (right) annual India-specific BC and OC emissions for post-monsoon residue burning season (Oct + Nov) over 2003-2019.

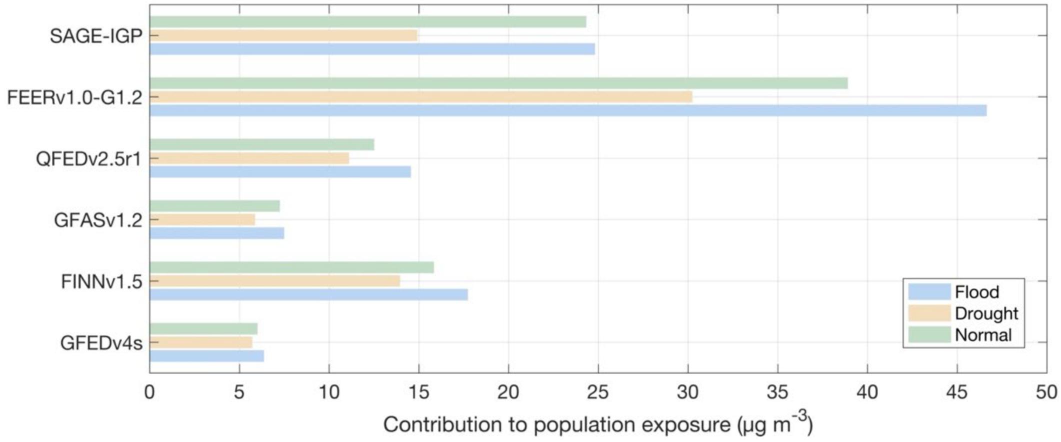

Figure 22. Contribution to annual population exposure to primary $PM_{2.5}$ (BC + OC) due to emissions in post-monsoon residue burning season (Oct + Nov) in flood, drought, normal years (based on Supplementary Fig. 7) by emissions inventory.

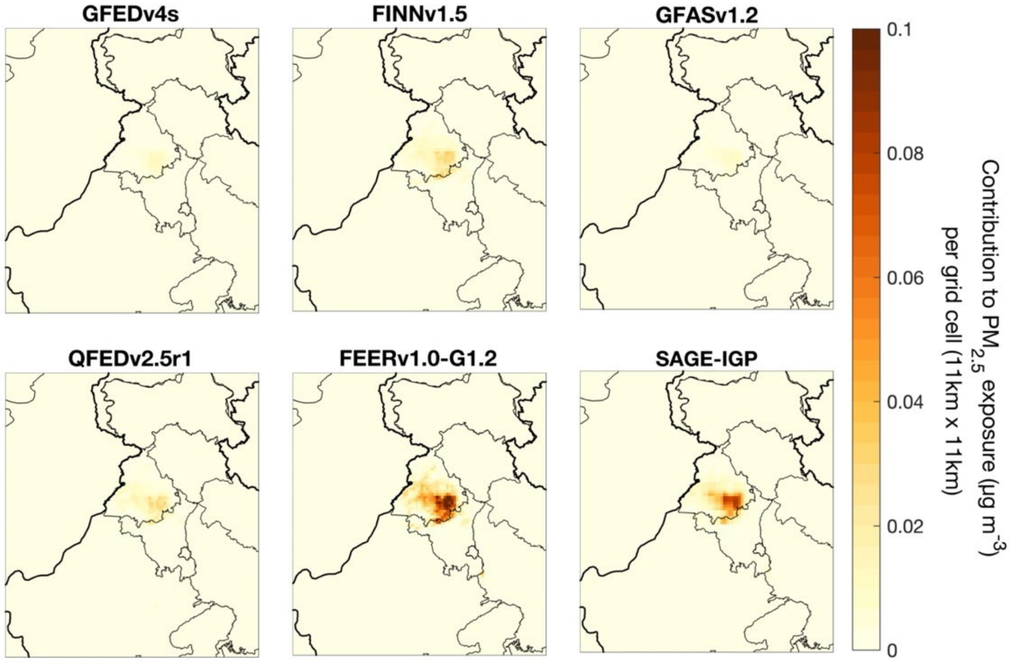

Figure 23. Average contribution to population exposure to primary $PM_{2.5}$ (BC + OC) due to emissions in rice residue burning season (Oct + Nov) by location and inventory. India administrative maps are obtained from the Database of Global Administrative Areas, Version 4.1 (https://gadm.org/data.html). Coast outlines are plotted based on data in MATLAB [32].

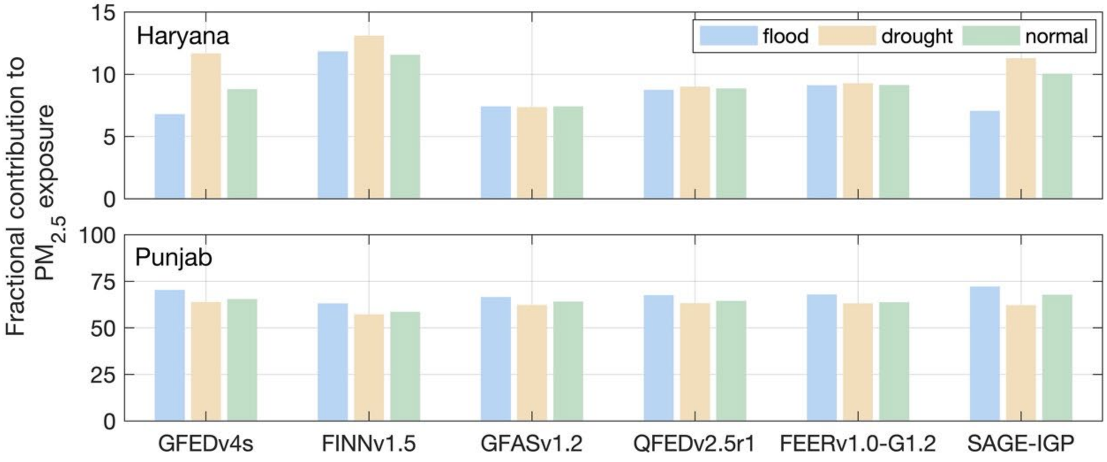

Figure 24. Fractional contribution to $PM_{2.5}$ exposure enhancement due to agricultural emissions in Haryana and Punjab compared with that due to total agricultural emissions in Oct-Nov in India.

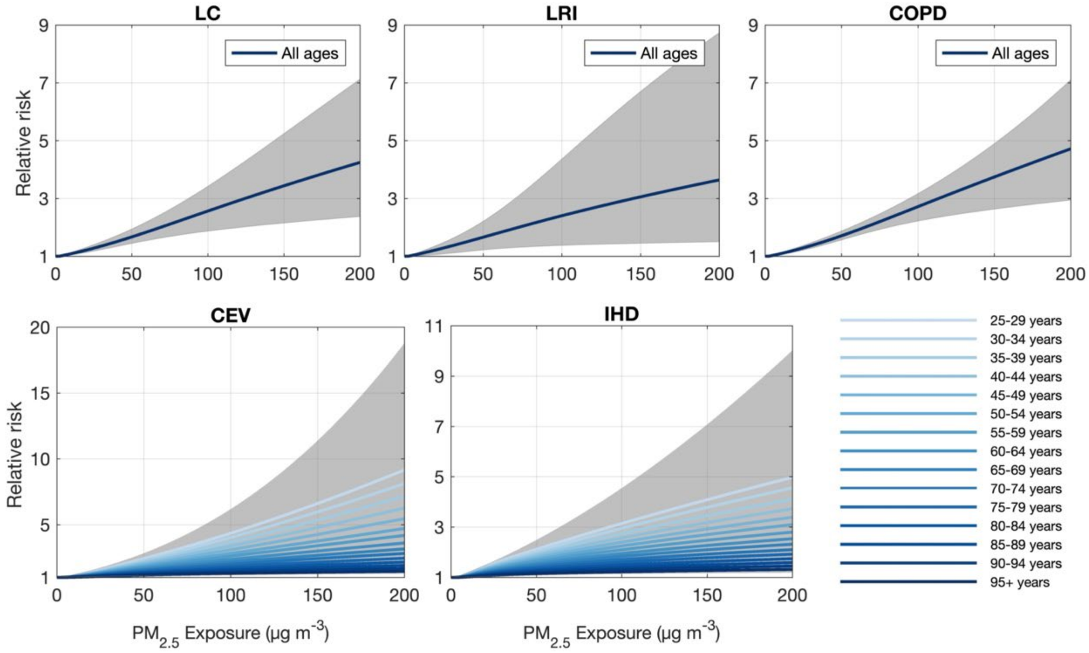

Figure 25. Relative risk curves based on GEMM for each cause of disease. The GEMM curves are obtained from Burnett et al., 2018 [21].

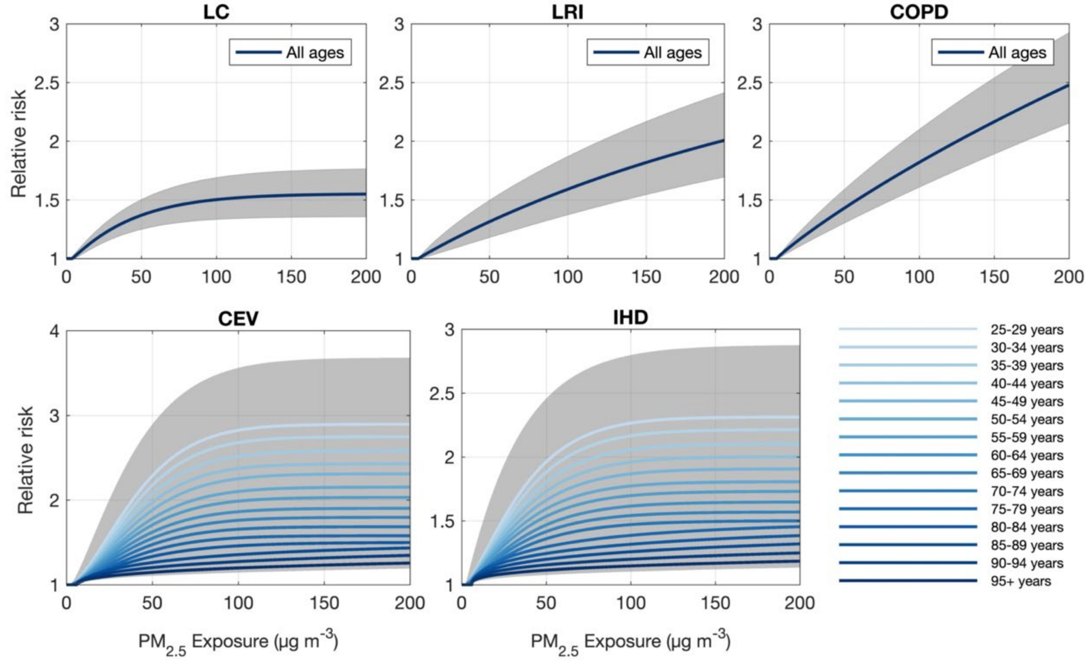

Figure 26. Relative risk curves based on MR-BRT for each cause of disease. The MR-BRT curves are obtained from https://ghdx.healthdata.org/record/ihme-data/global-burden-disease-study-2019-gbd-2019-particulate-matter-risk-curves.

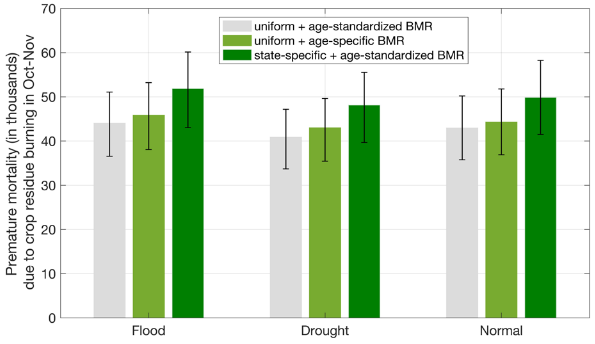

Figure 27. Annual premature mortality in India due to crop residue burning in Oct-Nov in flood, drought and normal years using different baseline mortality rates (BMR). Uniform + age-standardized BMR and Uniform + age-specific BMR are obtained from GBD 2019 (http://ghdx.healthdata.org/gbd-results-tool), state-specific + age-standardized BMR is obtained from Chowdhury and Dey 2016 [31].

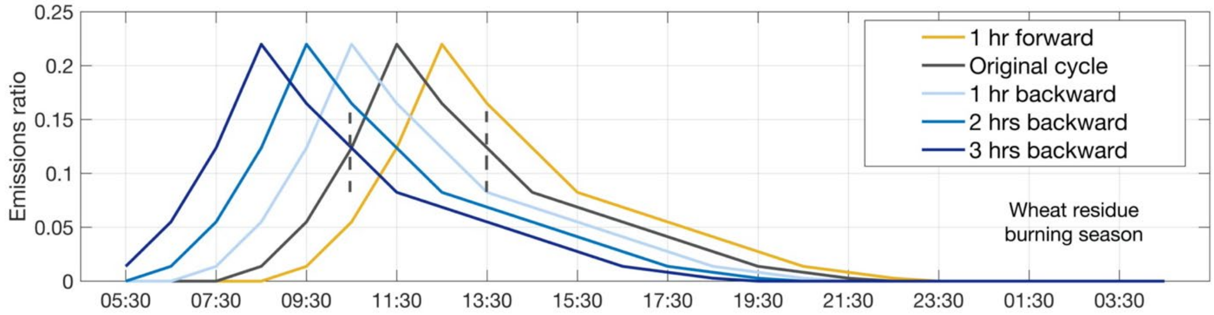

Figure 28. Diurnal cycles of local agricultural burning emissions for wheat residue burning season where emissions at 13:30 LT are around same as those at 10:30 local time (LT), with a peak at 11:30 LT, based on MODIS fire detection analysis by Vadrevu et al., 2011, 2013 [32,34], which shows relatively consistent release of fires (Terra/Aqua ~ 0.8-1.0) in wheat residue burning season, because farmers tend to start burning earlier when it is cooler in the morning. Since the fires can extend into night and burning in the dry season tends to have a longer cycle, we also assume here a longer and smoother tail of the diurnal cycle.

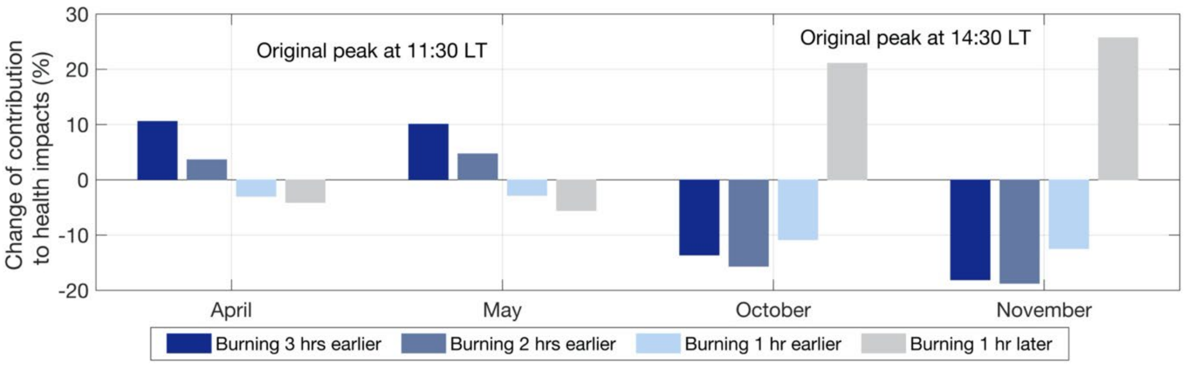

Figure 29. Similar to Figure 5 in the main text, but with original burning peak (diurnal cycle) for wheat residue burning based on Supplementary Fig. 28.

Compared with the original diurnal cycle in Supplementary Fig. 4, we find less improvement during wheat residue burning season using a different diurnal cycle. The choice of diurnal cycles may be arbitrary, and the impact attribution is sensitive to the assumptions we make. But the broad assumption that burning earlier brings benefit remains, since the peak is now set at 11:30 LT, when the boundary layer height (BLH) is approaching the peak. And with this diurnal cycle, burning 1 hour later (12:30 LT) gives the largest benefit, since it corresponds to the case "burning 2 hours earlier". Burning 2 and 3 hours earlier increases the contribution to impacts, probably because the BLH is not high enough in the early morning to help pollutants disperse, corresponding to the cases "burning 4 hours earlier" and "burning 5 hours earlier" in Figure 5 in the main text. Thus, burning activities should preferably peak between 10:00-12:00 to maximize the cost savings from burning.

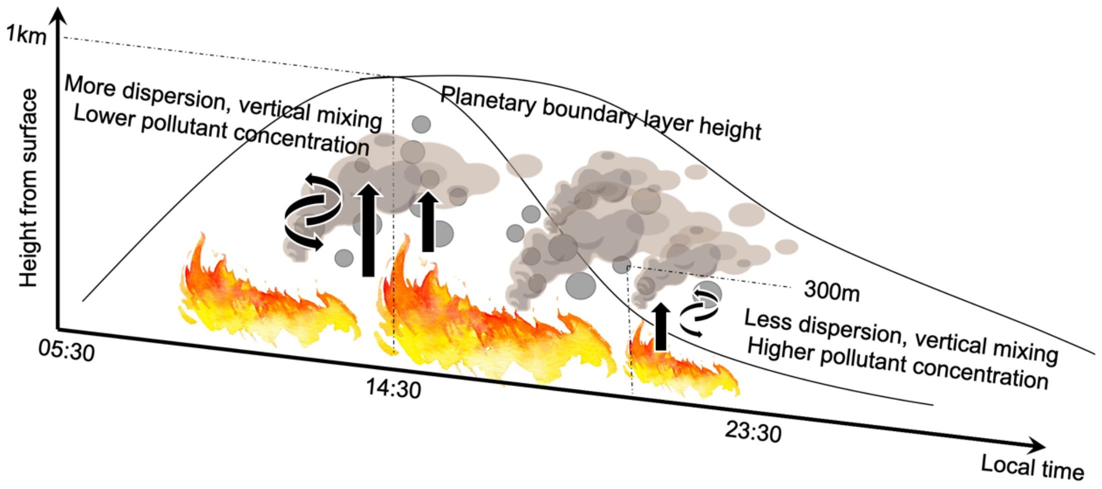

Figure 30. A schematic figure of diurnal changes of planetary boundary layer height and its impact on pollutants from ground sources (e.g. burning of crop residue).

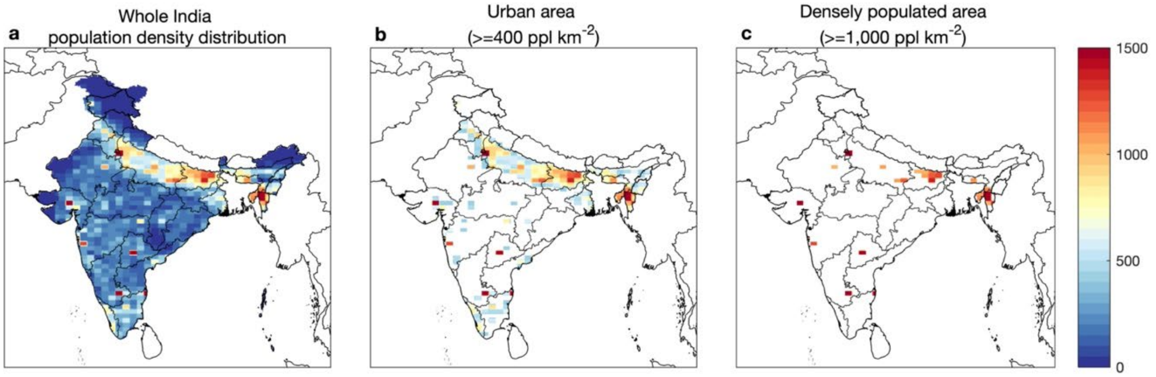

Figure 31. Population density map of India in people per square kilometer (ppl km^{-2}) corresponding to three target areas in adjoint simulations in Supplementary Fig. 1. Gridded population data in India is obtained from https://sedac.ciesin.columbia.edu/data/set/gpw-v4-population-count-rev11. India administrative maps are obtained from the Database of Global Administrative Areas, Version 4.1 (https://gadm.org/data.html). Coast outlines are plotted based on data in MATLAB [32].

Supplementary References

1. Hammer, M. S., A. van Donkelaar, C. Li, A. Lyapustin, A. M. Sayer, N. C. Hsu, R. C. Levy, M. J. Garay, O. V. Kalashnikova, R. A. Kahn, M. Brauer, J. S. Apte, D. K. Henze, L. Zhang, Q. Zhang, B. Ford, J. R. Pierce, and R. V. Martin. Global Annual $PM_{2.5}$ Grids from MODIS, MISR and SeaWiFS Aerosol Optical Depth (AOD), 1998-2019, V4.GL.03. Palisades, New York: NASA Socioeconomic Data and Applications Center (SEDAC). https://doi.org/10.7927/fx80-4n39 (2022).
2. Shi, Y., Matsunaga, T., Saito, M., Yamaguchi, Y. and Chen, X. Comparison of global inventories of CO2 emissions from biomass burning during 2002–2011 derived from multiple satellite products. *Environ. Pollut.* 206, 479-487 (2015).
3. Vijayakumar, K., Safai, P.D., Devara, P.C.S., Rao, S.V.B. and Jayasankar, C.K. Effects of agriculture crop residue burning on aerosol properties and long-range transport over northern India: A study using satellite data and model simulations. *Atmos. Res.* 178, 155-163 (2016).
4. Liu, T., Marlier, M. E., Karambelas, A., Jain, M., Singh, S., Singh, M. K., Gautam, R., and DeFries, R. S. Missing emissions from post- monsoon agricultural fires in northwestern India: regional limitations of MODIS burned area and active fire products. *Environ. Res.Commun.* 1, 011 007, (2019).
5. Cusworth, D. H., Mickley, L. J., Sulprizio, M. P., Liu, T., Marlier, M. E., DeFries, R. S., Guttikunda, S. K., and Gupta, P.: Quantifying the influence of agricultural fires in northwest India on urban air pollution in Delhi, India. *Environ. Res. Lett.* 13, 044 018, (2018).
6. Van Der Werf, G.R., Randerson, J.T., Giglio, L., Van Leeuwen, T.T., Chen, Y., Rogers, B.M., Mu, M., Van Marle, M.J., Morton, D.C., Collatz, G.J. and Yokelson, R.J. Global fire emissions estimates during 1997–2016. *Earth Sys. Sci. Data*. 9, 697-720 (2017).
7. Wiedinmyer, C., S. K. Akagi, Robert J. Yokelson, L. K. Emmons, J. A. Al-Saadi, J. J. Orlando, and A. J. Soja. The Fire INventory from NCAR (FINN): A high resolution global model to estimate the emissions from open burning. *Geosci. Model Dev.* 4, 625-641 (2011).
8. Kaiser, J.W., Heil, A., Andreae, M.O., Benedetti, A., Chubarova, N., Jones, L., Morcrette, J.J., Razinger, M., Schultz, M.G., Suttie, M. and Van Der Werf, G.R. Biomass burning emissions estimated with a global fire assimilation system based on observed fire radiative power. *Biogeosciences.* 9, 527-554 (2012).
9. Koster, R.D., Darmenov, A.S. and da Silva, A.M. The Quick Fire Emissions Dataset (QFED): Documentation of Versions 2.1, 2.2 and 2.4 (No. NASA/TM-2015-104606/Vol. 38) (2015).
10. Ichoku, C. and Ellison, L. Global top-down smoke-aerosol emissions estimation using satellite fire radiative power measurements. *Atmos. Chem. Phys.*14, 6643-6667 (2014).
11. Liu, T., Mickley, L.J., Singh, S., Jain, M., DeFries, R.S. and Marlier, M.E. Crop residue burning practices across north India inferred from household survey data: bridging gaps in satellite observations. *Atmos. Environ.: X.* 8, 100091 (2020).
12. Liu, T., Mickley, L.J., Marlier, M.E., DeFries, R.S., Khan, M.F., Latif, M.T. and Karambelas, A. Diagnosing spatial biases and uncertainties in global fire emissions inventories: Indonesia as regional case study. *Remote Sens. Environ.* 237, 111557 (2020).
13. Koplitz, S.N., Mickley, L.J., Marlier, M.E., Buonocore, J.J., Kim, P.S., Liu, T., Sulprizio, M.P., DeFries, R.S., Jacob, D.J., Schwartz, J. and Pongsiri, M. Public health impacts of the severe haze in Equatorial Asia in September–October 2015: demonstration of a new framework for informing fire management strategies to reduce downwind smoke exposure. *Environ. Res. Lett.* 11, 094023 (2016).
14. GBD MAPS Working Group. Burden of Disease Attributable to Major Air Pollution Sources in India. Special Report 21., Health Effects Institute. https://www.healtheffects.org/publication/gbd-air-pollution-india (2018)
15. Giles, D.M., Holben, B.N., Tripathi, S.N., Eck, T.F., Newcomb, W.W., Slutsker, I., Dickerson, R.R., Thompson, A.M., Mattoo, S., Wang, S.H. and Singh, R.P. Aerosol properties over the Indo-Gangetic Plain: A mesoscale perspective from the TIGERZ experiment. *J. Geophys. Res. Atmos.* 116, 18 (2011).
16. Kurokawa, J., Ohara, T., Morikawa, T., Hanayama, S., Janssens-Maenhout, G., Fukui, T., Kawashima, K. and Akimoto, H. Emissions of air pollutants and greenhouse gases over Asian regions during 2000–2008: Regional Emission inventory in ASia (REAS) version 2. *Atmos. Chem. Phys.* 13, 11019-11058 (2013).
17. David, L. M., Ravishankara, A., Kodros, J. K., Pierce, J. R., Venkataraman, C., and Sadavarte, P.: Premature mortality due to $PM_{2.5}$ over India: Effect of atmospheric transport and anthropogenic emissions. *GeoHealth*. 3, 2–10, (2019).
18. Kumar, R., Ghude, S.D., Biswas, M., Jena, C., Alessandrini, S., Debnath, S., Kulkarni, S., Sperati, S., Soni, V.K., Nanjundiah, R.S. and Rajeevan, M. Enhancing accuracy of air quality and temperature forecasts during paddy crop residue burning season in Delhi via chemical data assimilation. *J. Geophys. Res. Atmos.* 125, e2020JD033019 (2020).
19. Burnett, R. and Cohen, A., Relative risk functions for estimating excess mortality attributable to outdoor $PM_{2.5}$ air pollution: evolution and state-of-the-art. *Atmosphere*. 11, 589 (2020).
20. U.S. EPA. Regulatory Impact Analysis for the Final Revisions to the National Ambient Air Quality Standards for Particulate Matter; Office of Air Quality Planning and Standards, Health and Environmental Impacts Division: Research Triangle Park, NC, USA (2012).

21. Burnett, R., Chen, H., Szyszkowicz, M., Fann, N., Hubbell, B., Pope Iii, C.A., Apte, J.S., Brauer, M., Cohen, A., Weichenthal, S. and Coggins, J. Global estimates of mortality associated with long-term exposure to outdoor fine particulate matter, *Proc. Natl. Acad. Sci. U.S.A.* 115, 9592–9597 (2018).

22. Global Burden of Disease Collaborative Network. Global Burden of Disease Study 2017 (GBD 2017) Burden by Risk 1990-2017. Seattle, United States of America: Institute for Health Metrics and Evaluation (IHME) https://ghdx.healthdata.org/record/ihme-data/gbd-2017-burden-risk-1990-2017 (2018).

23. Murray, C.J., Aravkin, A.Y., Zheng, P., Abbafati, C., Abbas, K.M., Abbasi-Kangevari, M., Abd-Allah, F., Abdelalim, A., Abdollahi, M., Abdollahpour, I. and Abegaz, K.H. Global burden of 87 risk factors in 204 countries and territories, 1990–2019: a systematic analysis for the Global Burden of Disease Study 2019. *Lancet.* 396, 1223-1249 (2020).

24. Kollanus, V., Prank, M., Gens, A., Soares, J., Vira, J., Kukkonen, J., Sofiev, M., Salonen, R.O. and Lanki, T. Mortality due to vegetation fire-originated PM2.5 exposure in Europe-assessment for the years 2005 and 2008. *Environ. Health Perspect.*125, 30-37 (2017).

25. Kiely, L., Spracklen, D.V., Wiedinmyer, C., Conibear, L.A., Reddington, C.L., Arnold, S.R., Knote, C., Khan, M.F., Latif, M.T., Syaufina, L. and Adrianto, H.A. Air quality and health impacts of vegetation and peat fires in Equatorial Asia during 2004–2015. *Environ. Res. Lett*. 15, 094054 (2020).

26. Nawaz, M.O. and Henze, D.K. Premature deaths in Brazil associated with long-term exposure to $PM_{2.5}$ from Amazon fires between 2016 and 2019. *GeoHealth*, 4, 2020GH000268 (2020).

27. Ghude, S. D., Chate, D., Jena, C., Beig, G., Kumar, R., Barth, M., Pfister, G., Fadnavis, S., and Pithani, P. Premature mortality in India due to $PM_{2.5}$ and ozone exposure. *Geophys. Res. Lett.* 43, 4650–4658 (2016).

28. Bikkina, S., Andersson, A., Kirillova, E.N., Holmstrand, H., Tiwari, S., Srivastava, A.K., Bisht, D.S. and Gustafsson, Ö. Air quality in megacity Delhi affected by countryside biomass burning. *Nat.Sustain*. 2, 200-205 (2019).

29. Atkinson, R.W., Kang, S., Anderson, H.R., Mills, I.C. and Walton, H.A. Epidemiological time series studies of $PM_{2.5}$ and daily mortality and hospital admissions: a systematic review and meta-analysis. *Thorax.* 69, 660-665 (2014).

30. Global Burden of Disease Collaborative Network. Global Burden of Disease Study 2019 (GBD 2019) Particulate Matter Risk Curves. Seattle, United States of America: Institute for Health Metrics and Evaluation (IHME), https://ghdx.healthdata.org/record/ihme-data/global-burden-disease-study-2019-gbd-2019-particulate-matter-risk-curves (2021).

31. Chowdhury, S. and Dey, S. Cause-specific premature death from ambient $PM_{2.5}$ exposure in India: Estimate adjusted for baseline mortality, *Environ. Int.* 91, 283–290 (2016).

32. MATLAB version R2016b (9.1.0441655) Natick, Massachusetts: The MathWorks Inc. (2016).

33. Vadrevu, K. P., Ellicott, E., Badarinath, K., and Vermote, E. MODIS derived fire characteristics and aerosol optical depth variations during the agricultural residue burning season, north India. *Environ. Polluti.* 159, 1560–1569 (2011).

34. Vadrevu, K. P., Giglio, L., and Justice, C. Satellite based analysis of fire–carbon monoxide relationships from forest and agricultural residue burning (2003–2011). *Atmos. Environ.* 64, 179–191 (2013).
