# Supplementary material for: Air quality impacts of crop residue burning in India and mitigation alternatives
Source: Nat Commun. 2022 Nov 14;13:6537. doi: 10.1038/s41467-022-34093-z (PMC9663555; doi:10.1038/s41467-022-34093-z)
Supplement: Supplementary file 2 — Description of Additional Supplementary Files [file 41467_2022_34093_MOESM2_ESM.pdf]

File name: Supplementary Data 1
Description: Elemental analysis of crop residue by crop. Data adapted from Praspaliauskas, M., et al. "Study of chemical composition of agricultural residues from various agro-mass types." Biomass Conversion and Biorefinery 10.4 (2020): 937-948.

File name: Supplementary Data 2
Description: Population exposure to $PM_{2.5}$ attributable to agricultural residue burning by year and relative contribution by state.

File name: Supplementary Data 3
Description: Air quality impacts attributable to agricultural residue burning by state, season, and year.

File name: Supplementary Data 4
Description: Air quality impacts ($PM_{2.5}$ exposure, premature mortality, monetized cost) attributable to agricultural residue burning by year and total health expenditure, gross value added from agriculture in India. Total health expense and gross value added from agriculture are obtained from World Bank (https://data.worldbank.org/indicator).

File name: Supplementary Data 5
Description: Mean bias between GEOS-Chem simulated $PM_{2.5}$ and MODIS satellite-based $PM_{2.5}$ by state.

File name: Supplementary Data 6
Description: Model-observation comparison of daily PM_{25} at 34 cities in 2018 and 76 cities in 2019 (after data quality control), consistent with Supplementary Figure 15.

File name: Supplementary Data 7
Description: Comparison of daily population weighted $PM_{2.5}$ exposure due to all biomass burning emissions between forward and adjoint models from April 15-May 15 (S1-S3) and October 15-November 15 (S4-S6) in 2009. S1-S6 denote scenarios 1-6 (Supplementary Figure 16).

File name: Supplementary Data 8
Description: The estimated annual mortalities (in thousands) by cause of death in India attributed to exposure to $PM_{2.5}$ from agricultural burning by year.

File name: Supplementary Data 9
Description: Attributable air quality impacts (μg m^{-3}, thousand premature deaths) to crop residue burning for post-2015 period.

File name: Supplementary Data 10
Description: Number of detected crop residue burning events from 2016 to 2019 post-monsoon residue burning season. Data obtained from Consortium for Research Agroecosystem Monitoring & Modeling from Space, India Agricultural Research Institute (http://creams.iari.res.in/cms2/index.php).

File name: Supplementary Data 11
Description: Dry matter burned emissions (million tons of carbon) from GFED4.1s for post-2015 period.
